# Increased OXPHOS activity precedes rise in glycolytic rate in H-RasV12/E1A transformed fibroblasts that develop a Warburg phenotype

**DOI:** 10.1186/1476-4598-8-54

**Published:** 2009-07-31

**Authors:** Ad JC de Groof, Mariska M te Lindert, Michiel MT van Dommelen, Min Wu, Marieke Willemse, Amy L Smift, Mike Winer, Frank Oerlemans, Helma Pluk, Jack AM Fransen, Bé Wieringa

**Affiliations:** 1 Department of Cell Biology, Nijmegen Centre for Molecular Life Sciences, Radboud University Medical Centre, Nijmegen, The Netherlands; 2 Seahorse Bioscience, 16 Esquire Road, North Billerica, MA 01862, USA

## Abstract

**Background:**

The Warburg phenotype in cancer cells has been long recognized, but there is still limited insight in the consecutive metabolic alterations that characterize its establishment. We obtained better understanding of the coupling between metabolism and malignant transformation by studying mouse embryonic fibroblast-derived cells with loss-of-senescence or H-RasV12/E1A-transformed phenotypes at different stages of oncogenic progression.

**Results:**

Spontaneous immortalization or induction of senescence-bypass had only marginal effects on metabolic profiles and viability. In contrast, H-RasV12/E1A transformation initially caused a steep increase in oxygen consumption and superoxide production, accompanied by massive cell death. During prolonged culture *in vitro*, cell growth rate increased gradually, along with tumor forming potential in *in vitro *anchorage-independent growth assays and *in vivo *tumor formation assays in immuno-deficient mice. Notably, glucose-to-lactic acid flux increased with passage number, while cellular oxygen consumption decreased. This conversion in metabolic properties was associated with a change in mitochondrial NAD^+^/NADH redox, indicative of decreased mitochondrial tricarboxic acid cycle and OXPHOS activity.

**Conclusion:**

The high rate of oxidative metabolism in newly transformed cells is in marked contrast with the high glycolytic rate in cells in the later tumor stage. In our experimental system, with cells growing under ambient oxygen conditions in nutrient-rich media, the shift towards this Warburg phenotype occurred as a step-wise adaptation process associated with augmented tumorigenic capacity and improved survival characteristics of the transformed cells. We hypothesize that early-transformed cells, which potentially serve as founders for new tumor masses may escape therapies aimed at metabolic inhibition of tumors with a fully developed Warburg phenotype.

## Background

Carcinogenesis is a multi-step process that not only involves activation of oncogenes and/or inactivation of tumor suppressor genes [1], but also alterations in energy metabolism. Growth and viability of malignant cells is strongly dependent on their ability to adopt an altered metabolic profile that fulfills the synthetic and bioenergetic requirements for fast and uncontrolled growth. This metabolic phenotype is characterized by a shift from oxidative phosphorylation (OXPHOS) towards aerobic glycolysis as the main source of ATP production, a phenomenon first described by Otto Warburg [2].

The increased dependency of cancer cells on aerobic glycolysis is a well-recognized hallmark [3], but still relatively little is known about the factors that are in control during the early phases of the transformation process. Also, the issue whether or not this metabolic phenotype is an absolute prerequisite for advanced cancer progression is subject for debate [4]. Carbohydrate metabolism must warrant sufficient supply of ATP for cell growth, but also maximize macromolecular biosynthesis [5]. Intriguingly, cancer cells develop the ability to escape from senescence and apoptosis, processes in which glycolysis and mitochondrial pathways are actively engaged [6]. Furthermore, tumor cells maximize antioxidant capacity via the glutathione system, which is coupled to increased flux through the glycolysis-coupled pentose phosphate pathway (PPP) [7]. The question why tumor cells have a relatively high glycolytic rate compared to OXPHOS activity therefore goes well beyond the basics of which of the two processes is more efficient in terms of ATP production.

Carbohydrate metabolism has been studied extensively in the context of oncogene-induced cellular signaling, as mutations in metabolic enzyme-encoding genes themselves are rarely the direct cause of cancer [8]. For example, increased glycolysis is triggered by activation of the PI3K/AKT signaling pathway [5,9] or the p53 pathway [10]. An important aspect that has been less well studied is how metabolism changes dynamically in a transforming cell population. The Warburg phenotype in solid tumors *in vivo *is thought to be the result of multiple adaptive changes in metabolism in response to both cell-internal and -external cues. Hence the conversion from a dysplasic cell towards a full-blown cancerous state [11] is not just caused by genetic changes alone.

The step-wise development and associated metabolic adjustments of patient tumor cells is difficult to monitor, as it is virtually impossible to obtain a population of founder cells from which the tumor originally evolved. We therefore developed populations of mouse embryonic fibroblasts (MEFs) to investigate how adaptations in glycolytic-mitochondrial capacity are accomplished during oncogenic transition. Specifically, we studied cells in an immortalized state or directly after oncogenic transformation by mutant H-RasV12 and E1A. These latter cells were followed during the step-wise conversion to a fully cancerous state *in vitro *and passage through immune-deficient mice *in vivo*, which gave us the opportunity to analyze metabolism in a broad window of tumor stages. We focused on the sequential order in which adaptations in glycolytic-mitochondrial capacity were accomplished under constant extrinsic conditions of culture medium and oxygenation.

Our data show a sharp increase in oxygen consumption and a high rate of formation of reactive oxygen species (ROS) directly after H-RasV12/E1A transformation, with a smooth transition towards higher glycolytic rates later during tumorigenic development. We hypothesize that the immediate increase in oxygen consumption and OXPHOS activity sets a new and delicate balance in cell fate specification, driving cells in an "adapt-or-perish" regime. Subsequent escape from this situation is associated with gain in survival characterized by a glycolytic switch even under ambient oxygen levels, an adaptation typical for the Warburg phenotype.

## Results

### Generation of cell populations

We set up a new cell model system for the study of metabolic characteristics of immortalized and tumorigenic cells, all derived from one defined and genetically stable founder cell population, mouse embryonic fibroblasts (Prim-MEF; Table 1). Immortalized cells fulfill one early hallmark of cancer, namely limitless replicative life span [1]. The spontaneously immortalized population (Imm-MEF) was established by passaging the pool of Prim-MEFs through the replication bottleneck [12]. This transition is likely associated with the random acquisition of one or more genetic alterations such as mutation of p53 or loss of p19ARF [13]. A second pool of cells with a senescence-bypass phenotype (TBX2-MEF), thus genetically distinct from the spontaneously immortalized pool, was generated by overexpression of TBX2 in Prim-MEF at passage 2.

**Table 1:** Characteristics of primary, immortalized and tumorigenic cells in the panel

**cell population**	**abbreviation**	**description**	**cell diameter (μm)**	**cell volume (pL)**	**number of colonies in soft agar**	**tumor size (mm^2^) in BALB/c nu/nu**	**cell doubling time (h)**
Primary MEF	Prim-MEF	Isolated from 12–14.5 day-old embyos	16.3 ± 1.0 (3)	2.6 ± 0.5 (3)	none	n.a.	20.5 ± 0.7 (6)
Immortalized MEF (spontaneous)	Imm-MEF	Senescence bypass (3T3 passaging)	16.5 ± 0.7 (3)	2.7 ± 0.3 (3)	none	n.a.	24.1 ± 1.1 (6)
Immortalized MEF (TBX2)	TBX2-MEF	Retroviral transducton TBX2 gene	15.5 ± 0.3 (4)	2.1 ± 0.1 (4)	none	n.a.	19.9 ± 0.9 (6)
RasV12/E1A MEF (low passage)	RAS-LP	Retroviral transducton E1A/RasV12	11.0 ± 1.0 ** (4)	0.82 ± 0.2 ** (4)	131 ± 68 (5)	131 ± 62 (9)	16.8 ± 1.6 ^# ^(6)
RasV12/E1A MEF (high passage)	RAS-HP	Retroviral transducton E1A/RasV12	10.1 ± 0.4 *** (4)	0.58 ± 0.1 *** (4)	880 ± 164 †† (5)	497 ± 182 † (9)	12.2 ± 0.7*** ‡ (8)
RasV12/E1A MEF (tumor)	Ras-TUM	RAS-HP passage in BALB/c nu/nu mice	10.5 ± 0.3 *** (3)	0.65 ± 0.1 *** (3)	705 ± 146 †† (7)	n.a.	15.0 ± 1.0 ** (7)

Cell diameter, doubling time, and *in vitro *and *in vivo *tumorigenic characteristics were analyzed. Values represent average ± SEM (n). Cell diameter and cell doubling time decrease significantly in H-RasV12/E1A-transformed cells. Primary and immortalized cells do not form tumors *in vitro *or *in vivo*, whereas tumorigenic capacity of H-RasV12/E1A-transformed cells increases with passage number.

^1^For cell diameter analysis, approximately 120 cells per population were analyzed in each of the independent experiments.

^2^Soft agar assays were carried out in duplicate in each of the independent experiments.

^3^*In vivo *tumor assays were conducted on a total of nine animals as described in Material and Methods.

^4^For cell growth analysis and cell doubling time calculation, at least six independent experiments, each in duplicate, were conducted. Cells were seeded at 128,000 (Prim-MEF, Imm-MEF, TBX2-MEF) or 200,000 (Ras-LP, -HP, -TUM) cells per well of a 6-well plate.

Statistics:

**: p < 0.01; ***: p < 0.001 compared to Prim-MEF (one-way ANOVA/Bonferroni)

‡: p < 0.05 compared to Ras-LP (one-way ANOVA/Bonferroni)

†: p < 0.05; ††: p < 0.01 compared to Ras-LP (Student's t-test)

#: p < 0.10 for comparison Prim-MEF – Ras-LP (Student's t-test)

Co-expression of mutant RasV12 and adenovirus E1A in primary mouse cells constitutes a model of multistep tumorigenesis for establishment of oncogenic cell populations [14]. We used this principle to establish MEF populations at different phases of oncogenic transition towards a malignant phenotype via retroviral transduction of *pLPC-E1A-ires-H-RasV12 *in Prim-MEF cells at passage 2. The resulting cell pool was studied (i) directly after selection as *in vitro *cultivated cells (Ras-*L*ow *P*assage: 5–10), (ii) after prolonged passage *in vitro *(Ras-*H*igh *P*assage: > 25) or (iii) after injection of Ras-HP (p25) cells subcutaneously in the flank of immune-deficient mice and re-establishment as tumor cell populations in *in vitro *culture (Ras-TUM; between +3 and +10 passages after re-culturing). Each population was propagated as a mixed pool of cells with distinct viral DNA integration sites in the genome, and possibly differences in expression levels of the transforming transgenes. Thus, we avoided clonal analyses and excluded the possibility that one or few selective integrations of the transgene would dominate the phenotypic outcome.

### Changes in cell morphology

Cell morphology in Prim-MEF, Imm-MEF and TBX2-MEF populations was largely similar, but introduction of H-RasV12/E1A induced prominent morphology changes and resulted in a marked reduction in cell diameter (Table 1; Additional file 1: Fig. S1; Fig. S2A). Notably, the cellular protein concentration (Additional file 1: Fig. S2B-C) was decreased in immortalized cells, while it tended to increase in H-RasV12/E1A transformed cells.

### Growth characteristics of transformed cells

**Figure 1:**
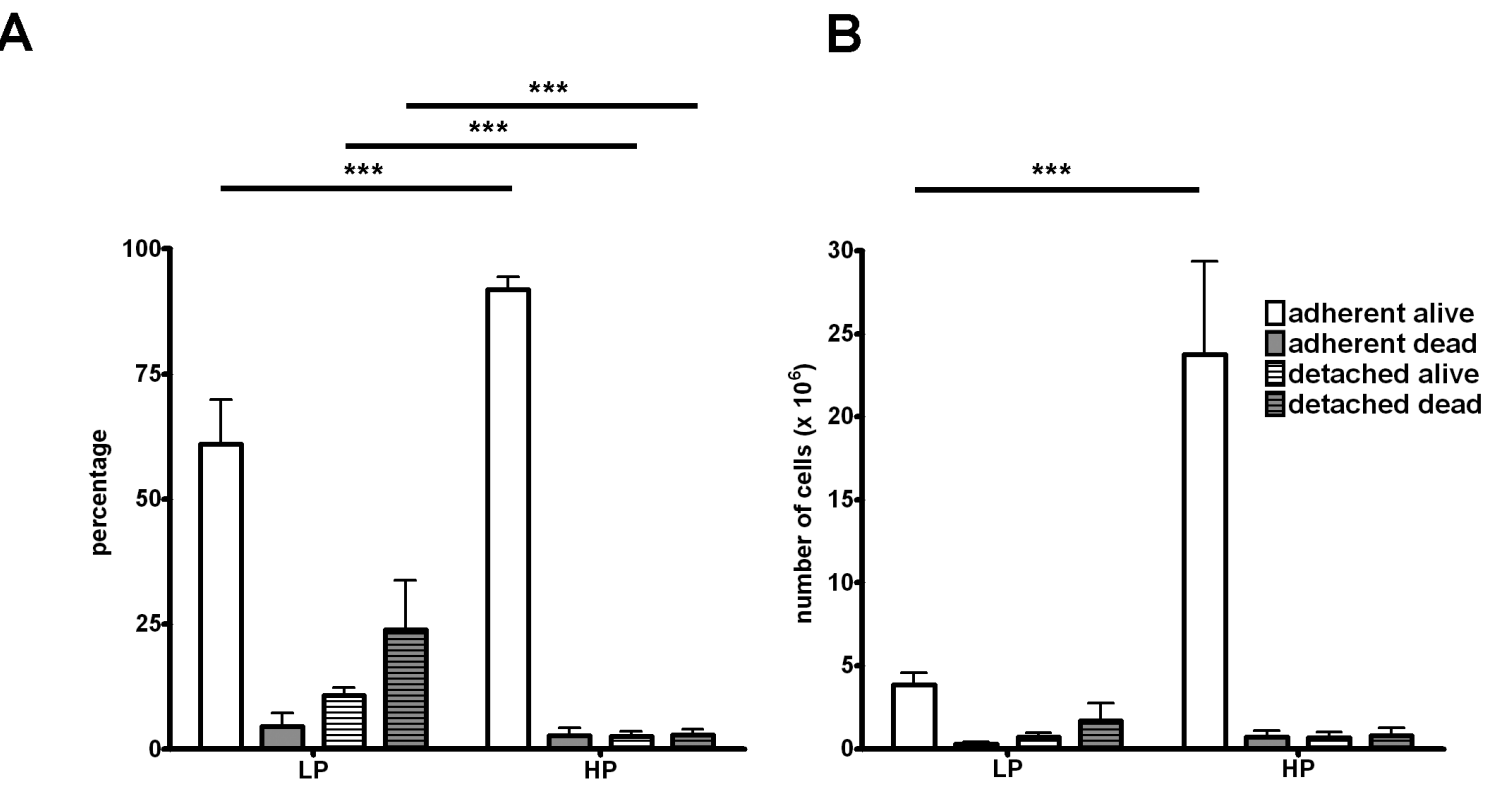
**Viability of Ras-LP and Ras-HP cells in standard culture conditions**. Adherent and non-adherent cells were harvested after 72 hours in culture, stained using tryphan-blue, and counted. **A**: Data presented as percentages of total amount of cells. ***: p < 0.001 (n = 5). Ras-LP cultures contain relatively more detached cells in the medium, of which the majority is dead. **B**: Data presented in (A) in absolute counts (n = 5). ***: p < 0.001 (n = 5). Ras-HP cell cultures contain relatively and absolutely more viable cells.

The effects of immortalization and H-RasV12/E1A expression on growth rate were studied. Cell doubling rates of Prim-MEF (between passage 3–8) and TBX2-MEF (between passage 15–25) did not differ (20 hrs; Table 1), but Imm-MEF (between passage 20–30) grew slower (+4 hrs). Doubling rate was reduced by three hours in Ras-LP cells, by eight hours in Ras-HP and by five hours in Ras-TUM cells. Our analyses revealed that cultures of Ras-LP cells contained a high percentage of detached, mostly dead, cells in the culture medium as assayed by tryphan blue staining (Fig. 1). This indicates that we underestimated the cell division time in these cells because of the reduced viability in the population. The transition from Ras-LP to Ras-HP was associated with a greatly improved survival chance and a drastic reduction in the amount of non-adherent cells in culture. We subsequently determined anchorage-independent growth characteristics of our cell populations in soft agar colony formation assays, and evaluated tumor forming potential in immune-deficient mice. After nine days, 5 to 7-fold more colonies had formed in Ras-HP and Ras-TUM soft agar assays compared to Ras-LP, whereas Imm-MEF, Prim-MEF and TBX2-MEF cultures gave no colonies at all (Table 1). Subcutaneous injection of cells in immune-deficient mice revealed that Ras-HP-derived tumors were four times larger than Ras-LP tumors after four weeks (Table 1). We conclude that the capacity of H-RasV12/E1A-transformed cells to form tumors increases with cell culture passage number.

**Figure 2:**
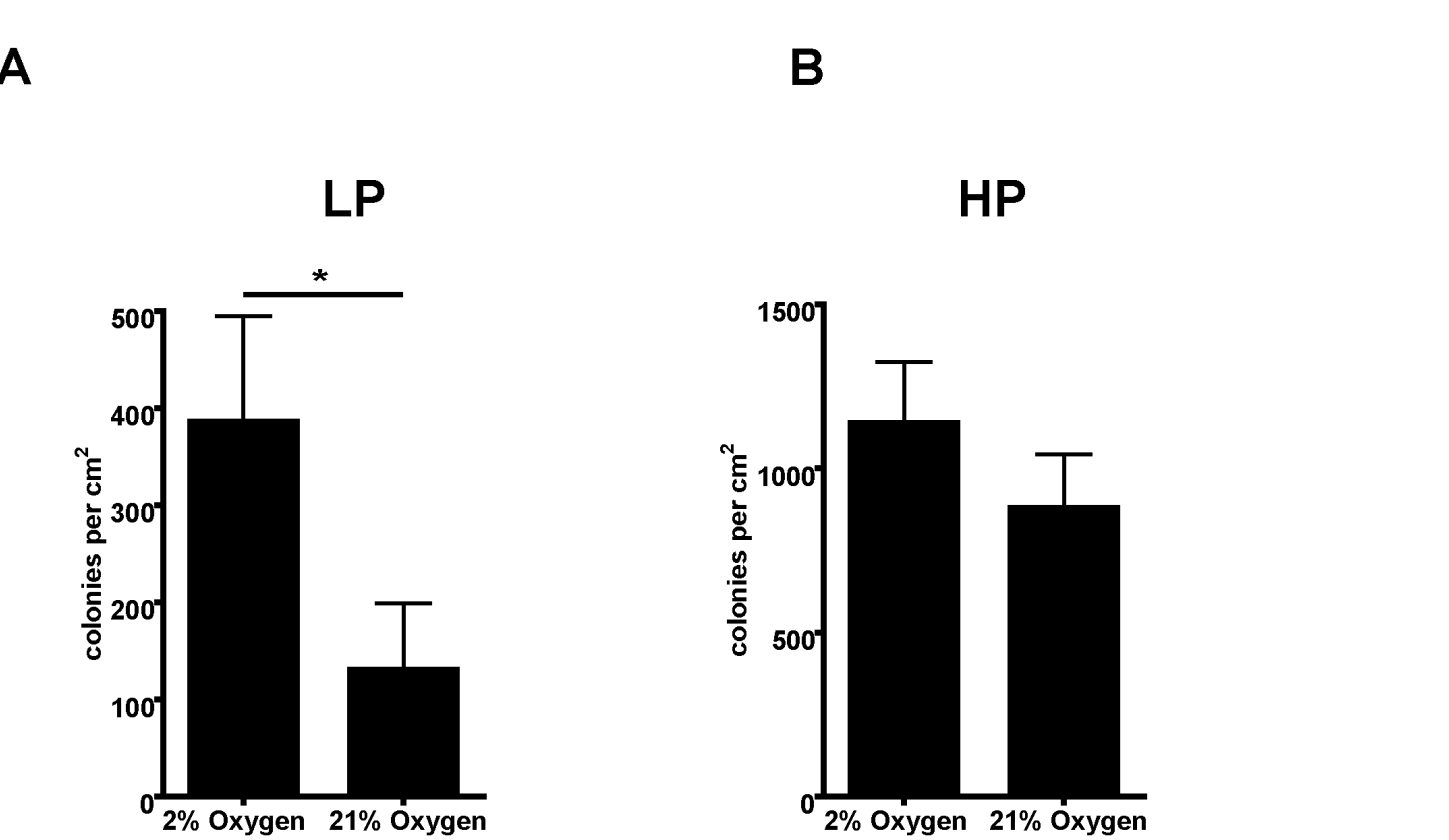
**Anchorage-independent growth in soft agar at different oxygen conditions**. A: Growth of Ras-LP cells in soft agarat 2% and 21% environmental oxygen. Lowering external oxygen levelsresults in a 3-fold increase in the number of colonies per cm^2^. *:p < 0.05 (Student's t-test). B: Growth of Ras-HP cells in soft agar at2% and 21% environmental oxygen. Lowering external oxygen levels inRas-HP soft agar cultures results in only a marginal 1.3-fold andnon-significant increase in colony number, showing that a glycolyticshift in these relatively glycolytic cells does not result in a largeincrease in viability and colony formation of cells in the soft agar.

Because all analyses were performed on cells growing at ambient oxygen condition in nutrient-rich culture media, we also analyzed the impact of low (2%) oxygen levels on cell growth and survival in soft agar, under conditions where diffusion limitation restricts nutrient supply. Under both high and low oxygen tension, the colony forming capacity of Ras-HP cells in soft agar was higher than that of Ras-LP cells. However, Ras-LP cells cultured at 2% oxygen formed 3-fold more colonies than at 21% oxygen, whereas Ras-HP formed only 1.3-fold more colonies at 2% oxygen (Fig. 2A–B). Ambient environmental oxygen supply as an extrinsic parameter thus plays an important negative role in fate specification of cells immediately after H-RasV12/E1A transformation.

### Lactic acid production in transformed cells

**Figure 3:**
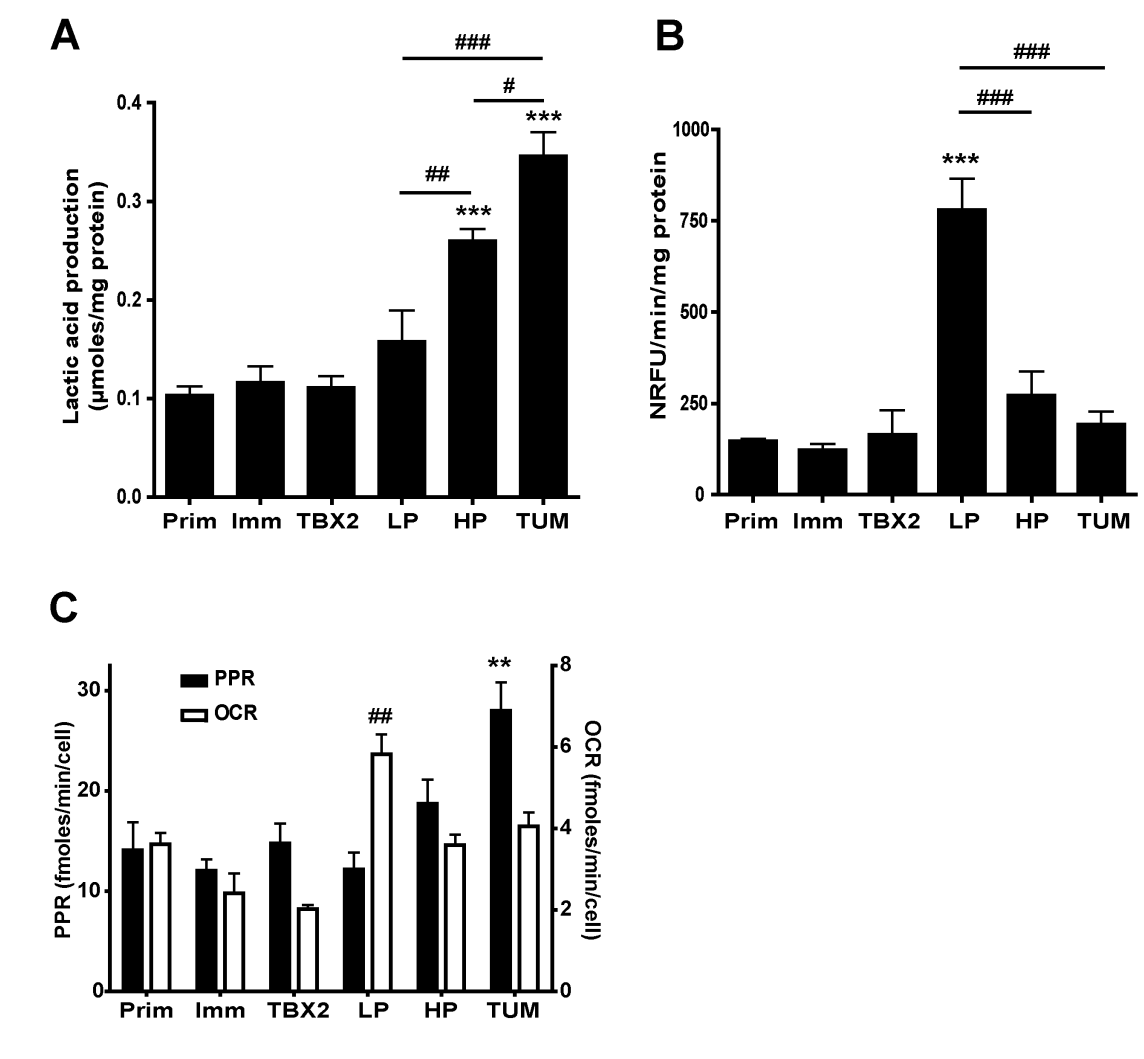
**Lactic acid production, oxygen consumption and proton production rate**. **A**: Lactic acid production in μ moles/mg protein (mean ± SEM). Lactic acid production was measured in media samples taken from the cell cultures used to obtain the cell doubling time data in Table 1. Six independent experiments, each in duplicate, were conducted for all cell populations, except Ras-HP (n = 8) and Ras-TUM (n = 7). Lactic acid production increases with passage number in H-RasV12/E1A-transformed cells. ***:p < 0.001 compared to Prim-MEF. #:p < 0.05; ##:p < 0.01; ###:p < 0.001 for Ras-LP – Ras-HP – Ras-TUM intercomparisons (one-way ANOVA/Bonferroni). **B**: Oxygen consumption in NRFU/min/mg protein measured in BD oxygen biosensor plates (mean ± SEM). Each experimental value represents n = 3 (Prim-MEF, Imm-MEF, TBX2-MEF) or n = 6 (other cell populations) independent assays, each carried out in triplicate. Cells were seeded at 200,000 cells per well of a 384-well plate. Oxygen consumption peaks immediately after H-RasV12/E1A transformation, and then decreases with passage number. ***:p < 0.001 compared to Prim-MEF. ###:p < 0.001 for Ras-LP – Ras-HP – Ras-TUM intercomparisons (one-way ANOVA/Bonferroni). **C**: Proton production rate (PPR) and oxygen consumption rate (OCR) per cell measured in Seahorse XF24 analysis 48 hours post seeding (mean ± SEM; n = 6, except Ras-HP, n = 4). Prim-MEF, Imm-MEF and TBX2-MEF cells were seeded at 25,000/well. Ras-LP, Ras-HP, Ras-TUM cells were seeded at 30,000/well. PPR: **:p < 0.01 compared to Prim-MEF. OCR: ##:p < 0.01 compared to Prim-MEF (one-way ANOVA/Bonferroni). These data confirm the gradual increase in cellular acidification and decrease in oxygen consumption described in (A) and (B) in real time analysis.

We further concentrated on the question which cell-intrinsic metabolic parameters play a role in H-RasV12/E1A-initiated tumor cell development. As increased lactic acid production is a hallmark change in intermediary metabolism of tumor cells [15], we determined lactic acid levels in culture medium at 24, 48 and 72 hours after seeding cells, alongside with cell counts (see above). This enabled calculation of lactic acid production per average cell in the time interval analyzed (Additional file 1: Fig. S3A). Because protein content per cell was not stable in cells of different size and volume (Additional file 1: Fig. S2B-C), we corrected for protein content per cell to enable accurate comparison between cells with different composition and morphology. The results presented in Fig. 3A show that lactic acid production in μ moles/mg protein were equal in Prim-MEF, Imm-MEF and TBX2-MEF, but increased 1.5-fold in Ras-LP cells, 2.5-fold in Ras-HP and 3.5-fold in Ras-TUM cells compared to Prim-MEF. Prolonged passaging of H-RasV12/E1A transformed cell populations induced a gradual shift towards a higher glycolytic rate.

### Steep increase in oxygen consumption rate immediately after transformation

The oxygen consumption rate was determined in normalized relative fluorescence units by analyzing trypsinized cells in BD Oxygen Biosensor multi-well plates, and subsequently corrected for protein content per cell (Fig. 3B; see Additional file 1: Fig. S3B). This parameter was relatively low in Prim-MEF and immortalized cell populations, but H-RasV12/E1A expression immediately induced a 5.6-fold increase in basal oxygen consumption rate per mg protein. Ras-HP cells showed 2.8-fold reduced oxygen consumption compared to Ras-LP, while the oxygen consumption of Ras-TUM cells was reverted to the level found in Prim-MEF cells.

Measurement of maximum oxygen consumption after uncoupling by FCCP revealed that mitochondria in Ras-LP cells function at 66% of their maximum respiratory capacity compared to only 25% in Prim-MEF and immortalized cells (Additional file 1: Fig. S4A-B). Oxygen consumption in all cell types was almost completely blocked by Complex V inhibitor oligomycin (Additional file 1: Fig. S4C), indicating that it was fully coupled to ATP production. Moreover, oxygen consumption was completely blocked by Complex III inhibitor myxothiazol and only marginally affected by extracellular supplementation of NADH, indicating that the vast majority of oxygen consumption occurred in mitochondria and was not due to membrane oxidase activity (Additional file 1: Fig. S4D), which significantly contributes to cellular oxygen consumption in a variety of tumor cell lines [16].

To verify these findings in an independent setting and to allow a direct calculation of fmoles oxygen consumed, we determined proton extrusion rate (indicative of anaerobic glycolytic rate) and oxygen consumption rate (indicative of mitochondrial OXPHOS) cells in adherent culture conditions using real time extracellular flux analysis [17]. In accordance with lactic acid production, the normalized proton production rate (PPR; Fig. 3C) per cell increased step-wise in the transition Prim-MEF – Ras-LP – Ras-HP – Ras-TUM. Analysis of oxygen consumption rate in adherent cells (OCR; Fig. 3C) in real time showed drastically increased oxygen consumption in Ras-LP cells compared to Prim-MEF, which again appeared diminished in Ras-HP and Ras-TUM. Thus, H-RasV12/E1A transformation directly induced high OXPHOS activity, which gradually diminished during prolonged culture *in vitro *and passage through immune-deficient mice.

### Expression levels of glycolytic and mitochondrial proteins

The shift in the balance between aerobic and anaerobic metabolism may be associated with abundance of several key enzymes in glycolytic and mitochondrial pathways, or by altered metabolite flux through these reactions [18]. Table 2 and Additional file 1 (Fig. S5A-B) show that levels of glycolytic pathway enzymes and mitochondrial enzymes were surprisingly constant (variations less than 50%) in all cell populations, suggesting that altered flux rates are responsible for the increased glycolytic flux in Ras-HP and Ras-TUM cells. We considered possible altered glycolytic flux regulation at the level of the LDH isoenzyme expression, but found only marginal changes in the LDH isoenzyme profile (data not shown).

**Table 2:** Protein expression levels in primary, immortalized and tumorigenic cell populations

	**Prim-MEF**	**Imm-MEF**	**TBX2-MEF**	**RAS-LP**	**RAS-HP**	**RAS-TUM**
**Glycolysis**						
Aldolase	1.0	1.1	1.1	1.0	1.0	1.1
GAPDH	1.0	0.9	1.0	1.4	1.4	**1.5**
Pyruvate kinase	1.0	0.8	0.8	1.0	1.1	1.0
LDH-A	1.0	0.8	0.8	1.0	1.1	1.2
**OXPHOS**						
Porin	1.0	1.3	1.3	1.4	0.9	1.0
B39	1.0	0.7	0.7	1.3	**1.5**	**1.5**
PDH-E2	1.0	1.0	1.2	1.2	1.0	1.2
PDH-E1a	1.0	1.0	1.1	1.3	1.4	1.2
**NAD metabolism**						
cMDH	1.0	0.6	1.2	**2.4**	**3.3**	**2.9**
mMDH	1.0	0.9	0.9	1.3	1.1	1.1
NAMPT*	nd	nd	nd	1.0	**1.9**	**2.6**

Semi-quantitative Western blot was performed as described in Material and Methods. Protein expression levels of Prim-MEF were arbitrarily set to 1.0. Glycolytic and mitochondrial enzymes do not show upregulation at the protein level in immortalized or transformed cell populations. Two enzymes involved in cellular NADH metabolism, MDH1 and NAMPT showed increased protein levels.

*Indexed on Ras-LP (1.0): protein levels in Prim-MEF were extremely low and could not be reliably quantified (nd: not detectable).

### Expression levels of proteins involved in cellular pyridine nucleotide balance

The setting of the cytosolic NAD^+^/NADH ratio determines the enzymatic rate of the GAPDH reaction, the LDH reaction, and the shuttling of NADH towards mitochondria via the malate-aspartate shuttle and is therewith an important determinant of pyruvate flux. We investigated if the alteration of flux through glycolytic and mitochondrial pathways was associated with changes in enzymes that determine the NAD^+^/NADH redox couple. Cytosolic malate dehydrogenase (MDH1) levels were two-fold higher in H-RasV12/E1A transformed than in non-transformed cells. In contrast, mitochondrial MDH (MDH2) levels appeared similar in all cell populations (Table 2; Additional file 1: Fig. S5C). Furthermore, the expression level of NAMPT, a key enzyme in the NAD^+ ^salvage pathway and one of the main regulators of total cellular NAD level, increased immediately after H-RasV12/E1A expression. The induction of MDH1 and NAMPT could have important implications for the pyridine nucleotide balance.

### Increased pyridine nucleotide levels in H-RasV12/E1A transformed cells

Steady state levels and redox state of pyridine nucleotides are important indicators and regulators for cell state and metabolic signaling activity [19]. We studied the pyridine nucleotide levels by HPLC to reveal if adjustments in the balance between glycolysis and OXPHOS were indeed reflected in changes in steady-state levels of total cellular ATP, NAD^+^, NADH, NADP, or NADPH (see Additional file 2; Additional file 1: Fig. S6). This analysis showed a 30–40% increase in total cellular NAD(H) (NAD^+ ^plus NADH) levels, but not NADP(H) (NADP^+ ^plus NADPH) levels, in RasV12/E1A transformed populations relative to unchanged total cellular ATP level. Unfortunately, neither of the two HPLC analysis methods used allowed reliable quantification of the NAD^+^/NADH or NADP^+^/NADPH redox status due to methodological constrains.

### Mitochondrial NAD^+^/NADH ratio is correlated with OXPHOS activity

**Figure 4:**
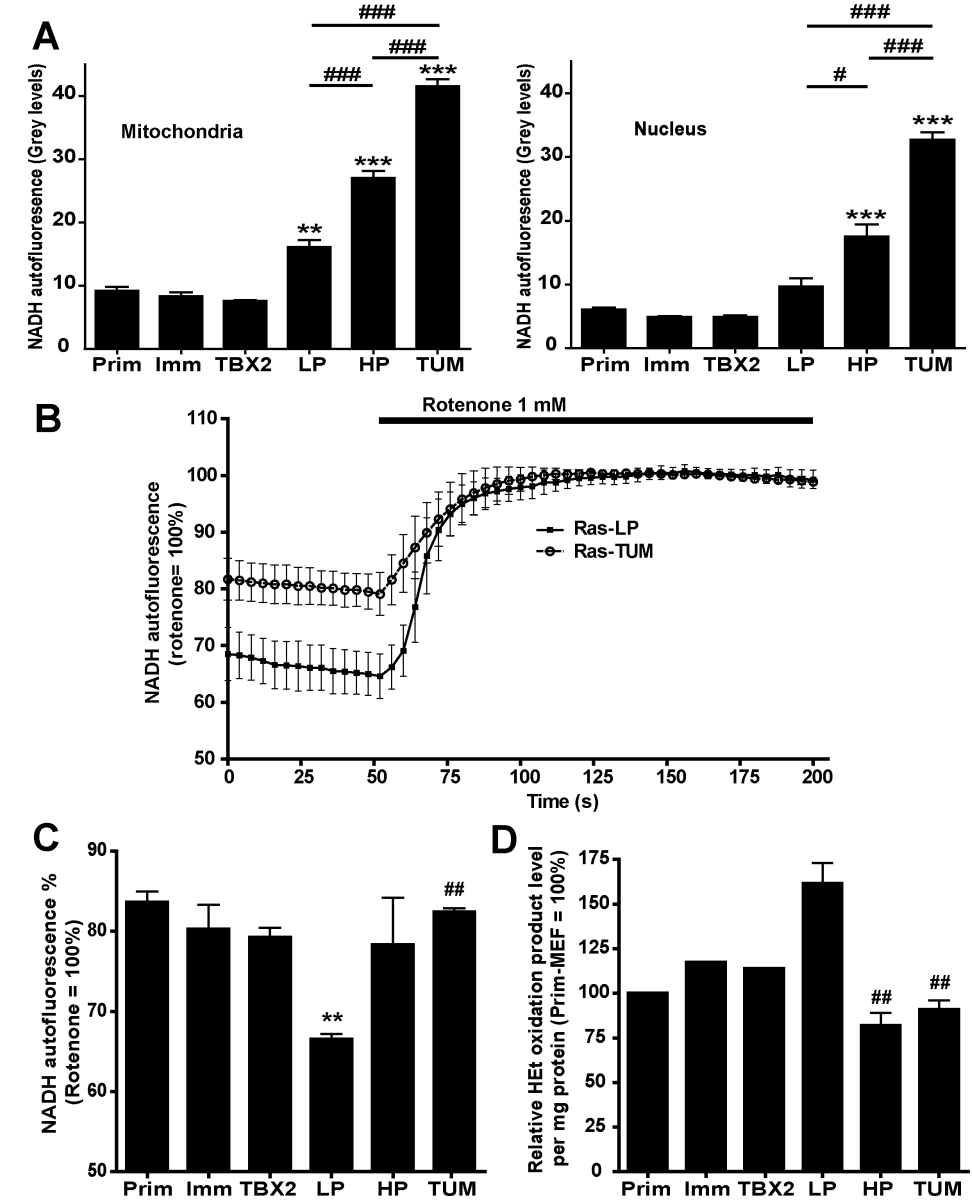
**Analysis of NADH autofluorescence**. **A**: Mitochondrial and nuclear NADH autofluorescence levels (mean ± SEM of a minimum of 3 independent experiments) expressed in absolute grey levels. Note that the signals were obtained from cells with clearly distinct morphology, which makes intercomparison of Prim-MEF, Imm-MEF and TBX2-MEF on the one hand, and Ras-transformed populations on the other, difficult. Absolute NADH autofluorescence increases gradually with passage number in H-RasV12/E1A-transformed cells. **B**: Representative recordings of NADH autofluorescence in Ras-LP and Ras-TUM cells before and after rotenone application. Rotenone inhibition of Complex I results in a differential increase in NADH autofluorescence. The percentage NADH autofluorescence is indicative of different rates of mitochondrial respiration. These data confirm the relatively higher respiration rates in Ras-LP cells. **C**: Cumulative data from n = 3 NADH autofluorescence/rotenone analyses carried out on separate days, with a minimum of 3 coverslips per day. Note that this parameter is cell morphology-independent. **:p < 0.01 compared to Prim-MEF ##:p < 0.01 for Ras-LP – Ras-TUM intercomparison (one-way ANOVA/Bonferroni). **D**: Superoxide levels expressed as fluorescence signal of HEt oxidation products per cell. Ras-LP levels were arbitrarily set at 100%. Oxygen consumption data per cell are available in Additional file 1, Fig. S4B and Fig. S4D. HEt fluorescence in Ras-LP, HP and TUM cells was determined in three independent experiments, each in duplicate, with at least 20,000 cells per assay. Prim-MEF, Imm-MEF and TBX2-MEF HEt fluorescence was determined in one experiment, in duplicate. Superoxide levels correlate with respiration rates measured in (B) and (C). ##:p < 0.01 compared to Ras-LP in a Student's t-test.

More detailed information on levels and compartmentalization of non-protein bound NAD(P)H was obtained by autofluorescence microscopy [20]. NAD(P)H signals, the majority of which is NADH, were largely confined to mitochondria and nucleus. Two to three-fold brighter autofluorescence signals were recorded from mitochondria and nuclei of cells in the Ras-HP and Ras-TUM pools (Fig. 4A), suggesting increased NAD(P)H levels in these populations. However, quantitative interpretation of this finding may be complicated by deviant morphological appearance of mitochondria in these two populations compared to Prim-MEF cells. We therefore studied the mitochondrial NAD^+^/NADH redox state specifically by concentrating on the relationship between autofluorescence levels and complex I (CI) activity in the electron transport chain (ETC) of the OXPHOS system, a parameter that is independent of cell morphology. Treatment with CI inhibitor rotenone blocks NADH consumption and elicits a direct increase in mitochondrial NADH level. Measurement of the difference in NADH fluorescence before and after rotenone administration thus is indicative for the fraction of cellular NADH that is actually used for turnover in OXPHOS activity. Fig. 4B and 4C convincingly show that the NADH-signal set point is at 66% of the maximum rotenone-value for Ras-LP, compared to 78–84% for all other cell populations. These results strongly suggest that Ras-LP cells have intrinsic properties that force them to use a larger fraction of their NADH content for flux through OXPHOS than Ras-HP or Ras-TUM cells, explaining the relatively high oxygen consumption of our Ras-LP cells. Ras-LP cells switched at passage six to galactose containing medium, a condition used to further maximize flux through mitochondrial OXPHOS [21], initially grew slower, and died around passage fourteen (data not shown). To us, these observations suggest that it is impossible to reach the high passage stage solely on aerobic metabolism. Ras-HP cells did not survive the switch to galactose containing medium and died within a few days, indicating that their aerobic metabolic capacity is not sufficient to meet cellular growth demands. As a final control for our assays, we forced use of mitochondrial OXPHOS in TBX2-MEF cells by subjecting them to growth on galactose/pyruvate medium for 6 days. This regime resulted in a 2.5-fold stimulation of the mitochondrial oxygen consumption rate (Additional file 1: Fig. S7A) and induced a decrease of the intramitochondrial NADH-signal set point from 85% to 65% of the maximal rotenone value (Additional file 1: Fig. S7B).

### Increased oxygen consumption is correlated to increased superoxide production

The increased oxygen consumption rate and the altered mitochondrial NAD(H)-redox setpoint in mitochondria prompted us to test if superoxide levels, as indicators of mitochondrial respiratory activity, were higher in Ras-LP cells. Superoxide levels per cell were monitored by HEt conversion to ethidium. Subsequent FACS analysis revealed two-fold higher levels in Ras-LP cells compared to Ras-HP and Ras-TUM cells on a per cell basis (Fig. 4D). Based on the absence of significant membrane oxidase activity (Additional file 1: Fig. S4D), we concluded that the majority of superoxide is generated in the mitochondria. In a control experiment, we showed that the switch to galactose in TBX2 cells has similar inducing effects on HEt fluorescence (Additional file 1: Fig. S7C).

## Discussion

We performed a comprehensive analysis of sequential metabolic changes that occur in a panel of cell populations with loss of cell-cycle arrest or increased tumorigenic potential. These analyses gave us new insight in the relation between oncogenic transformation and the development of the typical Warburg phenotype. The direct relatedness of the different cell pools – all pools were derived from the same primary MEFs ground state cell population – gave us a unique opportunity to do comparative profiling and highlight the early-late order of adaptive metabolic changes in energy metabolism that occur during solid tumor development.

Two genetically independent immortalization procedures had only minor effects on cell size, morphology, and carbohydrate metabolism, and yielded metabolic phenotypes with strong similarity to the original Prim-MEF cells. In contrast, H-RasV12/E1A transformation caused a prominent change from flattened to spherical appearance and smaller volume (see also [22]). Similar changes have been reported before for other transformed cell types [23,24].

One of the most conspicuous observations in our study was that the metabolic phenotype initially switched to aerobic, OXPHOS-based, immediately after transformation. The augmented OXPHOS rate in Ras-LP cells was associated with increased total cellular NAD(H) levels, an altered mitochondrial NAD^+^/NADH setpoint, increased oxygen consumption and increased superoxide production. We think that OXPHOS induction in early transformed cells may be a general concept that precedes the well-documented Warburg effect or shift to aerobic glycolysis. The initial switch to aerobic metabolism has also been reported after oncogene-mediated transformation of human mesenchymal stem cells [25], human epithelial cells [26,27] and human breast cancer cells [28]. At this stage it is, however difficult to draw direct comparison between our findings in mouse cells and findings in the different human systems, as panels with related human cells lines comparable to our panel do not exist or were generated via use of increasing numbers of oncogenes [27]. Future work, involving analyses of metabolic fate specification in panels with human cells from mesenchymal, epithelial, or progenitor-stem cell origin, will therefore be needed to verify the prediction of high OXPHOS activity in early transformed cells.

Importantly, our results indicate that environmental factors can exert strong influence on viability of tumor cells during the early phase of the transformation process. Anchorage-independent growth capacity of Ras-LP cells increased under low oxygen conditions, presumably by a direct suppressive effect on the high intrinsic and "unbalanced" mitochondrial activity or simply by better coupling of glycolytic-mitochondrial pathways. For better explanation and discrimination between these possibilities a detailed comparison of relative mitochondrial and glycolytic pathway activity in cells in colonies formed under ambient oxygen or in hypoxia would be imperative. Unfortunately, such pathway analysis on relatively small numbers of cells in soft agar *in situ *is currently technically not possible, so we have to leave this analysis for future work. If we may extrapolate these findings to the *in vivo *situation, we would predict that nutrient levels in the environment, including oxygen levels, may determine the fate of early dysplastic cells in tissues. In the light of our results, we predict that low oxygen or hypoxia would enhance their capacity to grow as tumors, as was already proposed in the model of Gatenby and Gillies [29].

The later trajectory of oncogenic transformation displayed by our cell panel involved a gradual shift towards higher anaerobic glycolysis rates over 20 passages *in vitro *and passage through immune-deficient mice, accompanied with clear gain in survival ability. We demonstrate here that the pools of cells that progress towards a tumorigenic phenotype go through a distinct series of adaptational changes. Although this metabolic rewiring may also be controlled by specific mutations in regulatory genes that become dominant in the entire pool, we consider it more likely that cell-intrinsic metabolic signaling mechanisms, acting within the windows set by the culture conditions (i.e. nutrient and O_2 _supply) used in our experiments, drive the gradual shift from OXPHOS to glycolysis. We draw this conclusion because we observed a similar stepwise drift in metabolic phenotype in a parallel series of cell populations that were independently derived from MEFs from mice with a combined defect in phosphotransfer enzymes CK-B and AK1 [30]. At this point it is important to note that this entire transition trajectory occurred in continuous presence of 21% environmental oxygen and under constant medium conditions. Strikingly, H-RasV12/E1A expression only marginally affected protein expression levels of glycolytic, TCA and OXPHOS enzymes at all stages after transformation, implying that altered flux rates dominate and underlie the observed metabolic effects. The shift in mitochondrial NAD^+^/NADH ratio that we observed is in support of this model, as this ratio sets the flux rate of key metabolic reactions.

Adaptive changes in mitochondrial function, with or without further genetic programming, may provide growth- and survival advantage by conferring resistance to apoptotic cell death [31]. Cells with electron flow through the ETC and enhanced glycolytic flux may perform best, as these are properties associated with reduced ROS production and increased ROS scavenging ability, respectively. ROS induced damage is a well-known factor in cell viability control [7]. Indeed, in the H-RasV12/E1A cells mitochondria are the primary ROS source, as we observed a direct relationship between oxygen consumption and superoxide production. Indirect support for this relationship was also provided by our finding on glucose-galactose shift effects in TBX2-MEF cells.

We would like to emphasize that mechanisms other than those based on mitochondria-derived ROS effects could also explain the apparent coupling between tumor progression and increased glycolytic rate. Firstly, the metabolic infrastructure of cells could have changed due to Ras-induced alterations in cell morphology and cytoarchitecture. Cell surface changes, cell clustering, attachment and movement are processes that are coupled to actin cytoskeleton rearrangements. Bereiter-Hahn and co-workers [32] showed that changes in energy metabolism may result from rearrangements of the F-actin network, and conversely, that energy demand of motile actin-based structures is supplied predominantly by ATP derived from glycolysis. At the same time, mitochondrial dynamics are determined by a complex interplay between the inner membrane-matrix complex and cytoskeletal elements outside, possibly via F-actin and dynamin-related protein DRP1 [33]. Upregulation of glycolysis or alteration of mitochondrial properties may thus be directly coupled to Ras-induced cytoskeletal remodeling, and fulfill new needs for development of anchorage independent growth, or other essential prerequisites for tumorigenic progression.

As another speculative possibility, there may also be a connection between cell cycle progression and the balance between glycolytic or oxidative metabolism. H-RasV12 induces cell cycle progression via enhanced G1 to S phase transition [34], and also the E1A oncoprotein induces S-phase via induction of p300/CBP and *c*-Myc [35]. Recently, it was reported that slowly growing yeast cells in a low glucose environment exhibit a periodic metabolic cycle that alternates between glycolysis and respiration, where the cell division cycle is constrained to the reductive non-respiratory phase of this cycle, with DNA replication taking place only during the glycolytic phase [36,37]. If extrapolation of the yeast model to the situation in mammalian cells is valid, our findings may suggest that glycolysis is the default metabolic pathway adapted by Ras-transformed cells with enhanced proliferation capacity.

Finally, our findings can also be explained by early alterations in NAD^+^/NADH setpoint, acting as driver for subsequent changes in tumorigenic conversion. We consider this an interesting possibility because we observed changes in set point level of NAD(H) (NAD^+ ^plus NADH) metabolites and upregulation of NAMPT protein levels as parallel immediate early effects of H-RasV12/E1A transformation in our RAS-LP-HP-TUM series. Van der Veer et al. [38] reported that overexpression of NAMPT results in increased life span and resistance to oxidative stress. Our data show that total cellular NAD(H) levels increase upon cellular transformation and may relate to the simultaneous upregulation of the NAD-salvaging enzyme NAMPT at the protein level. Evidence is now accumulating that NAD^+ ^levels, and/or redox state of the NAD^+^/NADH couple in cytosol, nucleus and mitochondria are key factors in transcriptional regulation [39,40], modulation of growth-differentiation decisions in dividing cells [41], and survival control [42]. Moreover, the need for NAD^+ ^or NADH as essential co-factors in regulation of metabolic flux rates is already long known. While we regard our work as a first proof-of-concept it is clear that more study into these regulatory circuits – including those in human cells of different tissue origin – is necessary to draw definite conclusions about the control over early and late events in the Warburg transition.

## Conclusion

We show that H-RasV12/E1A transformation of cells causes instantaneous and dramatic upregulation of mitochondrial ETC-OXPHOS activity, and not aerobic glycolysis. This brings newly transformed cells in a dangerous metabolic balance, reflected by a high rate of cell death. At this early stage, cells have a limited capacity for anchorage independent growth. Based on our findings we hypothesize that cells then traverse an "adapt-or-perish" period. Those that develop enhanced glycolysis and relatively low mitochondrial activity have increased survival chances, adapt further, and commonly develop the Warburg phenotype of tumorigenic cells. Our data predict that early-transformed cells, with high OXPHOS activity and the potential to serve as founders for new tumor masses, may be missed in positron emission tomography images or escape therapies aimed at metabolic inhibition of tumors with a fully developed Warburg phenotype.

## Methods

### Cell culture

For routine propagation, cells at all stages were kept under identical culture conditions with high nutrient and oxygen supply, with the exception of Ras-TUM cells, which were in an *in vivo *tumor environment for a four-week period. Cells were grown in DMEM (25 mM glucose, Invitrogen, Breda, The Netherlands) supplemented with 4 mM glutamine, 1 mM pyruvate and 10% Fetal Calf Serum (FCS, Greiner Bio-One, Alphen aan den Rijn, The Netherlands) in 21% environmental oxygen conditions or in 2% oxygen conditions where indicated. Primary MEFs were derived from E12.5–14 dpc mouse embryos [43] with a heterogeneous C57BL/6/129Ola background and frozen at passage 1. We subsequently derived all other cell populations from this pool of MEF cells as described in the Results section. In some experiments, cells were cultured in glucose-free DMEM (Invitrogen) supplemented with 10% dialyzed FBS and 10 mM galactose to stimulate oxidative phosphorylation [44]. Details on retroviral transduction are available in Additional file 2.

### Microscopy, cell morphology and cell size analysis

Protocols and equipment for microscopy and cell size analysis are available in the Additional file 2.

### Soft agar assay and injection of cells in immune-deficient mice

Procedures for analysis of anchorage-indepent growth characteristics and tumor growth in immune-deficient mice, adopted from Vasseur et al. (2003) [14], are described in Additional file 2.

### Growth curves and metabolic profiling

For simultaneous determination of cell growth rate and lactic acid production, cells were seeded in 6-well plates. Medium was harvested and cells were counted at 24 h, 48 h, and 72 h. Lactic acid levels were determined using the Amplex Red glucose/glucose oxidase kit (Molecular Probes, Leiden, The Netherlands), with lactate oxidase (Sigma-Aldrich) in the reaction instead of glucose oxidase. Growth curves, cell doubling time and average cell number per hour were calculated in the exponential growth phase (24–72 hrs) to obtain metabolite production/consumption per average cell in culture. These numbers were converted to metabolite production/consumption per mg.

### Oxygen consumption

Cells were trypsinized, counted, resuspended in culture medium and transferred to the BD Oxygen Biosensor 384 multi-well plates (Becton Dickinson, Erembodegem-Aalst, Belgium). Cellular oxygen consumption was determined in culture medium, and in medium supplemented with FCCP (2 μM), oligomycin (2.5 μM), myxothiazol (1 μM) or NADH (1 μM; all Sigma-Aldrich). Fluorescence (485 nm excitation, 612 nm emission) was recorded in a POLARstar Optima Fluorescence polarization plate reader (BMG Labtech, IJsselstein, The Netherlands) in 2-min intervals at 37°C for 4 hrs. The maximum slope of fluorescence units/min was calculated and converted into normalized relative fluorescence units (NRFU/min).

### Real-time metabolic analysis

Simultaneous multiparameter metabolic analysis of cell populations in culture was performed in the Seahorse XF24 extracellular flux analyzer (Seahorse Bioscience, Billerica, MA, USA) as described by Wu et al. [17]. All six cell lines were cultured in growth medium for 48 hours in plate before real-time metabolic analysis. At the start of the assay, growth medium was removed and replaced with DMEM medium containing 5% FBS (free of bicarbonate). Basal OCR (oxygen consumption rate) and PPR (proton production rate) of the cells were measured repeatedly in 30 minutes. After XF assay, cells were harvested by trypsin-EDTA treatment and counted on ViCell (Beckman Coulter, Fullerton, CA, USA). The number of cells per well were used to normalize OCR and PPR.

### Lysate preparation and Western blot

Semi-quantitative Western Blot analysis was performed as described by De Groof et al. [45]. Details are available in Additional file 2.

### HPLC

For HPLC analysis, six 10 cm dishes (Prim, Imm-MEF, TBX2-MEF) or 6-cm dishes (other cell lines) were cultured. In each experiment, three plates were used for cell counts; the other three for metabolite extraction using the sample preparation method of Fiehn (MeOH/CHCl_3 _extraction method, see Additional file 2). We repeated the entire experiment using a perchlorid acid extraction sample preparation method. This method allowed parallel protein quantification using the Pierce BCA kit (Pierce, Etten-Leur, The Netherlands). Further details of the HPLC instrumentation and the experimental procedures are available in Additional file 2.

### Microscopic measurement of NAD(P)H autofluorescence

NAD(P)H autofluorescence levels were measured in the experimental setup described by Verkaart et al. [46]. A detailed description is available in Additional file 2.

### Analysis of superoxide production

Cell cultures were incubated in HEPES-Tris medium containing 10 μM hydroethidine (HEt; Molecular Probes) for 10 min at 37°C [46]. The reaction was stopped by thorough washing of the cells with PBS to remove excess HEt. After trypsinization and fixation in 2% paraformaldehyde, cells were washed and transferred to FACS tubes. Ethidium fluorescence was elicited by excitation at 488 nm and the emission signal detected at 540–600 nm or > 670 nm using FACScan equipment (Becton Dickinson).

### Statistical analysis

Statistical analyses were performed by one-way ANOVA and Bonferroni's multiple comparisons test. Pairwise comparisons were made between Prim-MEF and all other cell populations, or between Ras-LP and the other two Ras-transformed populations. Other statistical analysis methods are mentioned in the text.

## Abbreviations

CI: Mitochondrial Complex I; ETC: Electron transport chain; FCCP: Carbonyl cyanide-p-trifluoromethoxyphenylhydrazone; GAPDH: Glyceraldehyde-3-phosphate dehydro-genase; HEt: Hydroethidine; LDH: Lactate dehydrogenase; MDH: Malate dehydrogenase; MEF: Mouse embryonic fibroblast; NAMPT: Nicotinamide mononucleotide phosphoribosyl transferase; NRFU: Normalized relative fluorescence units; OCR: Oxygen consumption rate; OXPHOS: Oxidative phosphorylation; PPP: Pentose phosphate pathway; PPR: Proton production rate; ROS: Reactive oxygen species.

## Competing interests

Min Wu, Amy Smift and Mike Winer are employed at Seahorse Biosciences.

## Authors' contributions

BW was responsible for project planning. AdG and BW conceived and designed the experiments. The cell populations were generated and maintained by MtL, MaW, MvD and AdG. Metabolic parameters were analyzed by MtL, MvD and MaW at the NCMLS, and by MiWi, AS and MiWu at Seahorse Biosciences (Fig. 3C). FO performed HPLC measurements. NADH autofluorescence microscopy was performed by MvD and JF. HP and JF performed SEM analysis. AdG, JF and BW analyzed the data and wrote the paper. All authors read and corrected the paper and added suggestions.

## Supplementary Material

Additional file 1 **Additional Figures 1–7**. File contains Supplemental Figures 1–7 as referred to in the main article. Click here for file

Additional file 2 **Additional Material and Methods, Results, Supplemental Figure Legends**. File contains supplemental Material and Methods and Results as referred to in the main article. Click here for file
