# Supplementary material for: Increased OXPHOS activity precedes rise in glycolytic rate in H-RasV12/E1A transformed fibroblasts that develop a Warburg phenotype
Source: Mol Cancer. 2009 Jul 31;8:54. doi: 10.1186/1476-4598-8-54 (PMC2734543; doi:10.1186/1476-4598-8-54)
Supplement: Additional file 1 — Additional Figures 1–7. File contains Supplemental Figures 1–7 as referred to in the main article. [file 1476-4598-8-54-S1.pdf]

Figure S1

A primary and immortalized cells

B H-RasV12/E1A-transformed cells

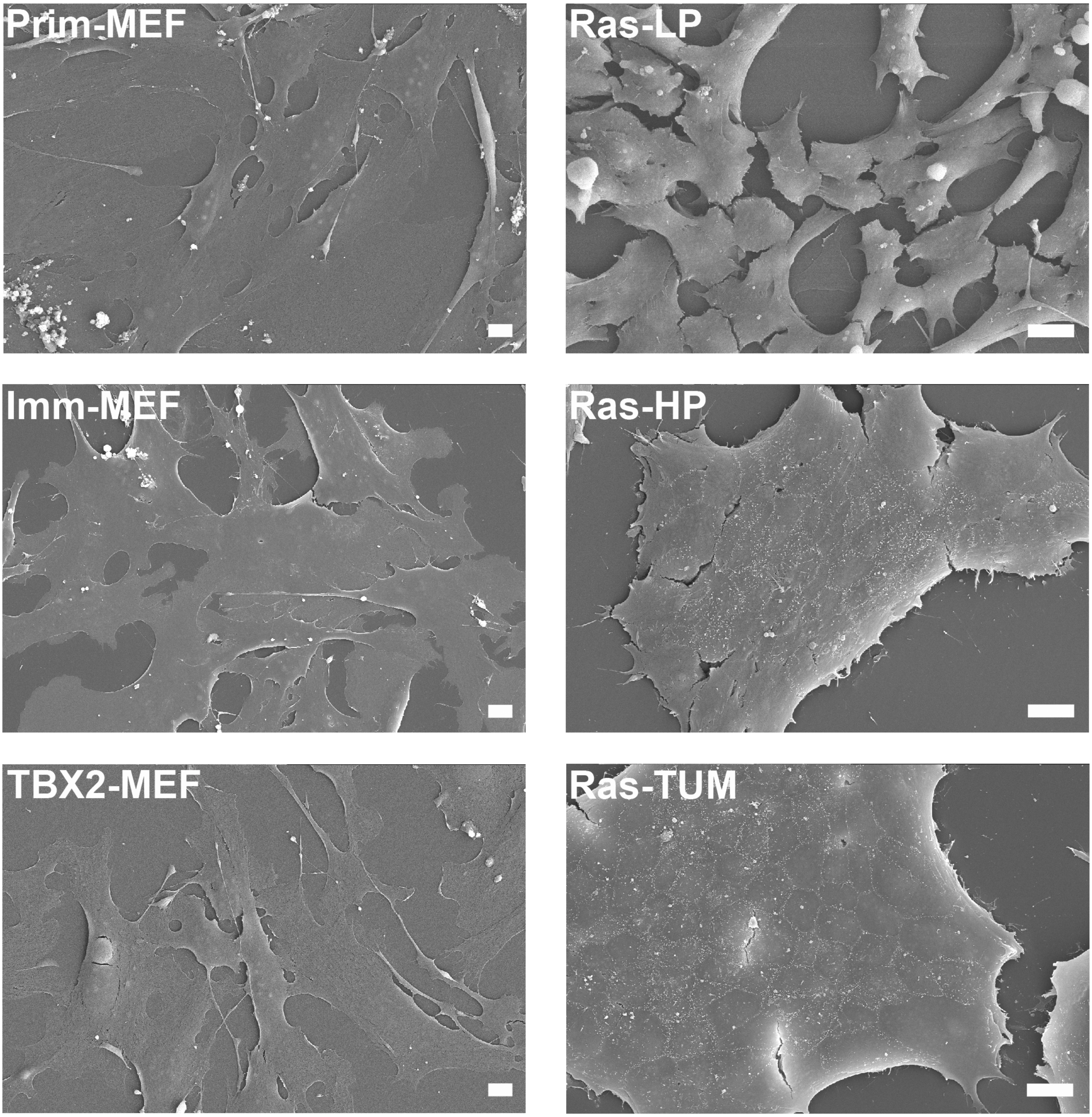

Figure S2

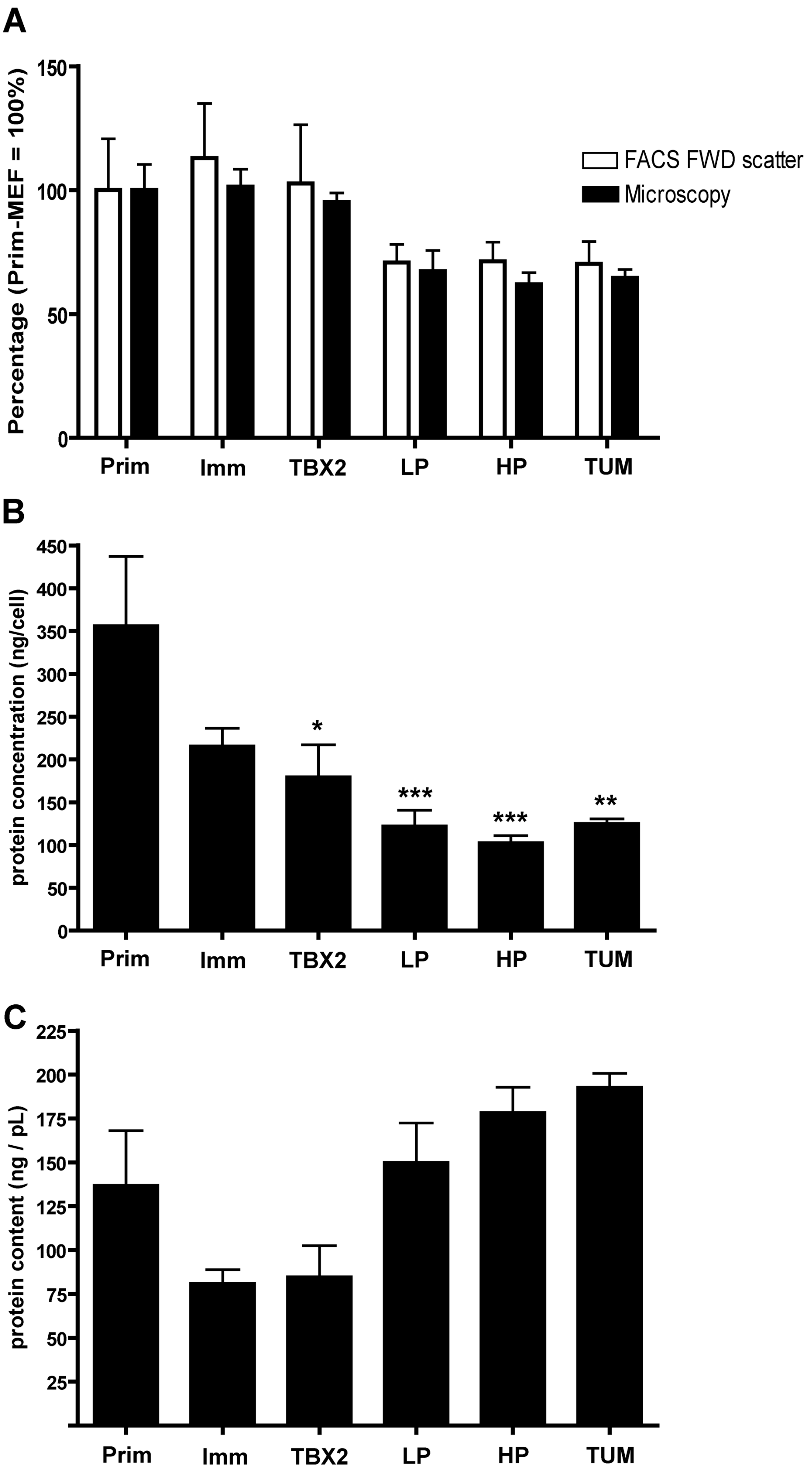
A
Percentage (Prim-MEF = 100%)
150
100
50
0
FACS FWD scatter
Microscopy
Prim
Imm
TBX2
LP
HP
TUM
B
protein concentration (ng/cell)
450
400
350
300
250
200
150
100
50
0
*
***
***
**
Prim
Imm
TBX2
LP
HP
TUM
C
protein content (ng / pL)
225
200
175
150
125
100
75
50
25
Prim
Imm
TBX2
LP
HP
TUM

Figure S3

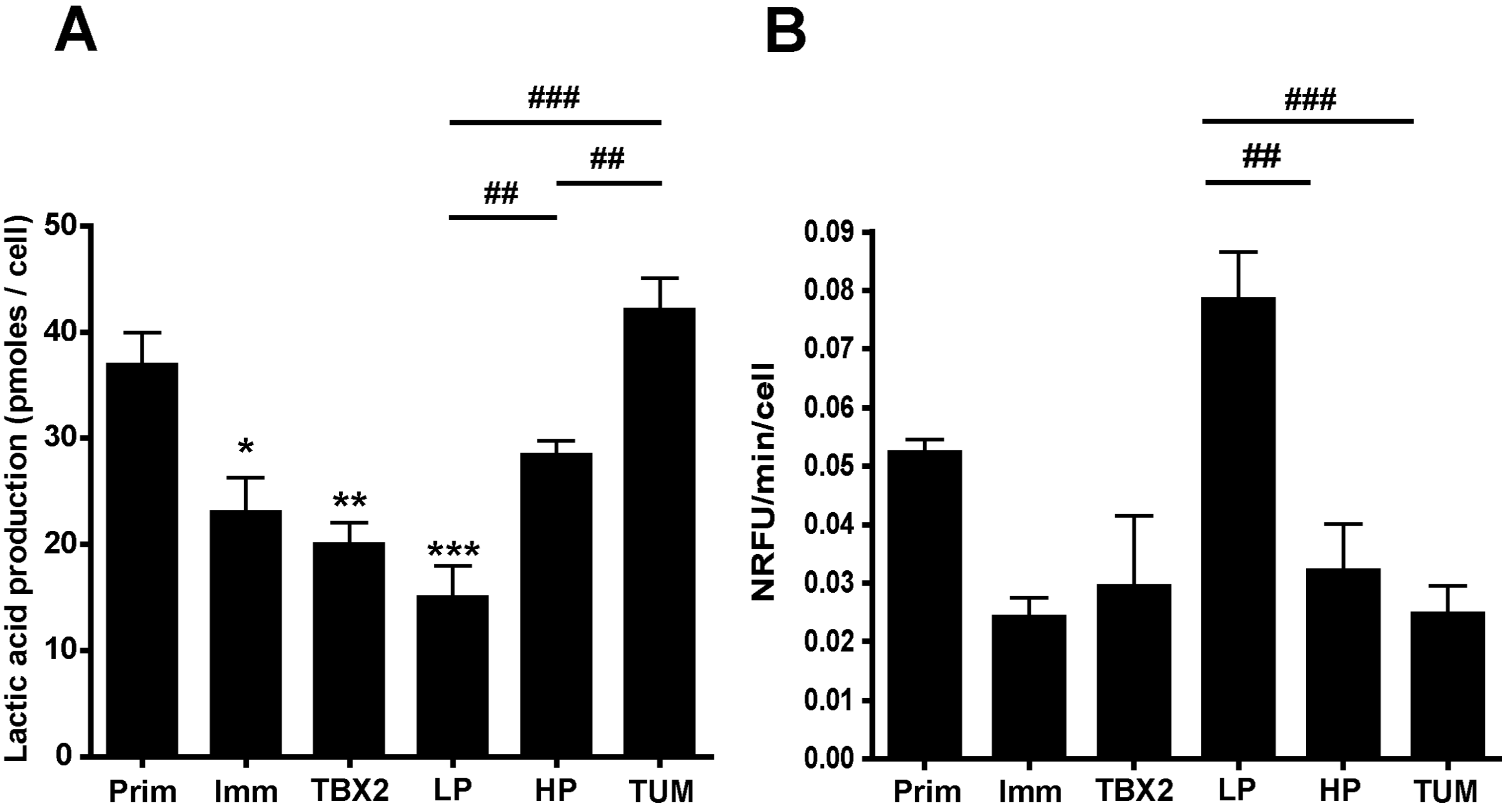
A
B
###
##
##
###
##
Lactic acid production (pmoles / cell)
50
40
30
20
10
0
*
**
***
Prim
Imm
TBX2
LP
HP
TUM
NRFU/min/cell
0.09
0.08
0.07
0.06
0.05
0.04
0.03
0.02
0.01
0.00
Prim
Imm
TBX2
LP
HP
TUM

Figure S4

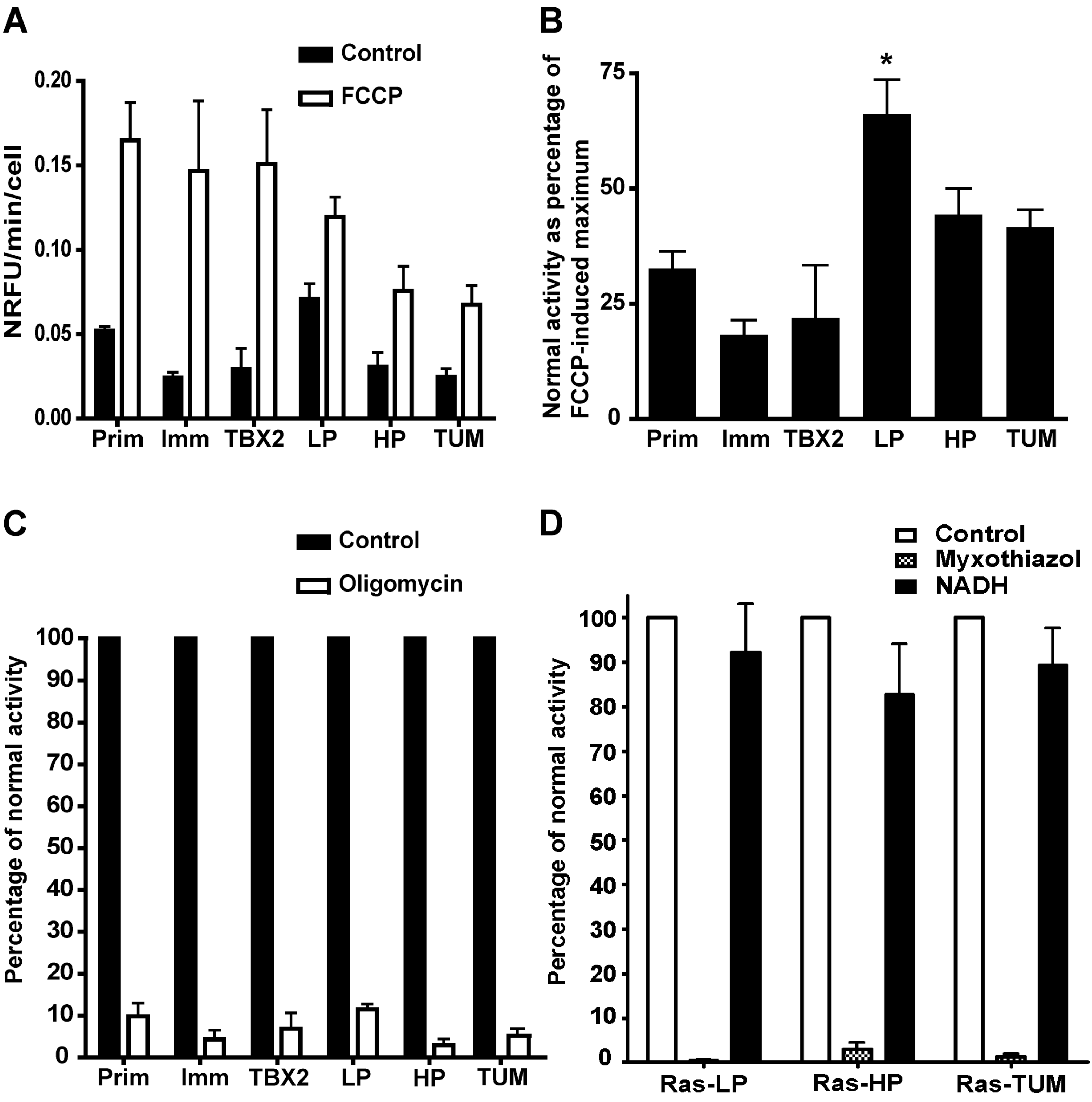
A
Control
FCCP
NRFU/min/cell
0.20
0.15
0.10
0.05
0.00
Prim
Imm
TBX2
LP
HP
TUM
B
Normal activity as percentage of FCCP-induced maximum
75
50
25
0
*
Prim
Imm
TBX2
LP
HP
TUM
C
Control
Oligomycin
Percentage of normal activity
100
90
80
70
60
50
40
30
20
10
0
Prim
Imm
TBX2
LP
HP
TUM
D
Control
Myxothiazol
NADH
Percentage of normal activity
100
90
80
70
60
50
40
30
20
10
0
Ras-LP
Ras-HP
Ras-TUM

Figure S5 (de Groof et al.)

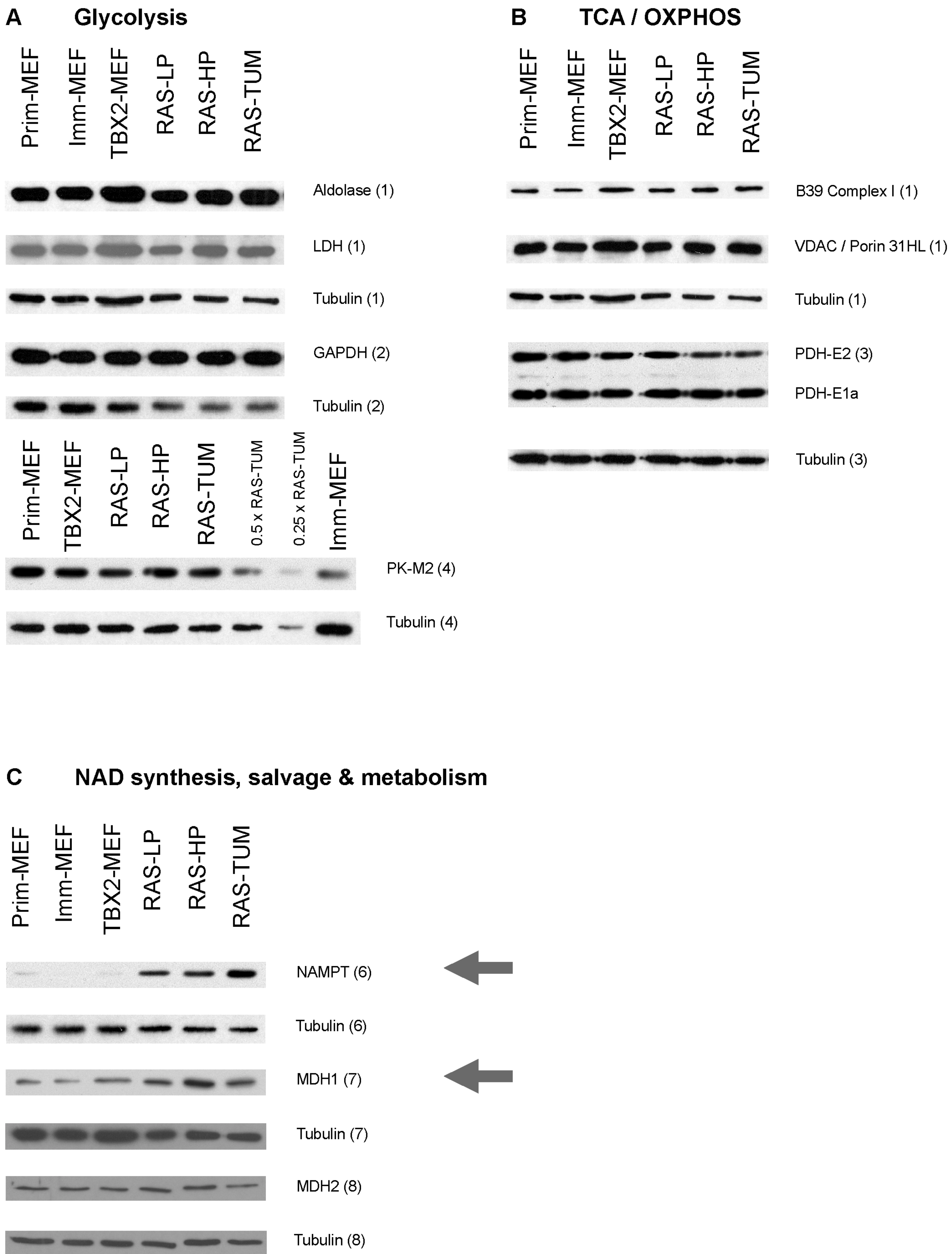

Figure S6

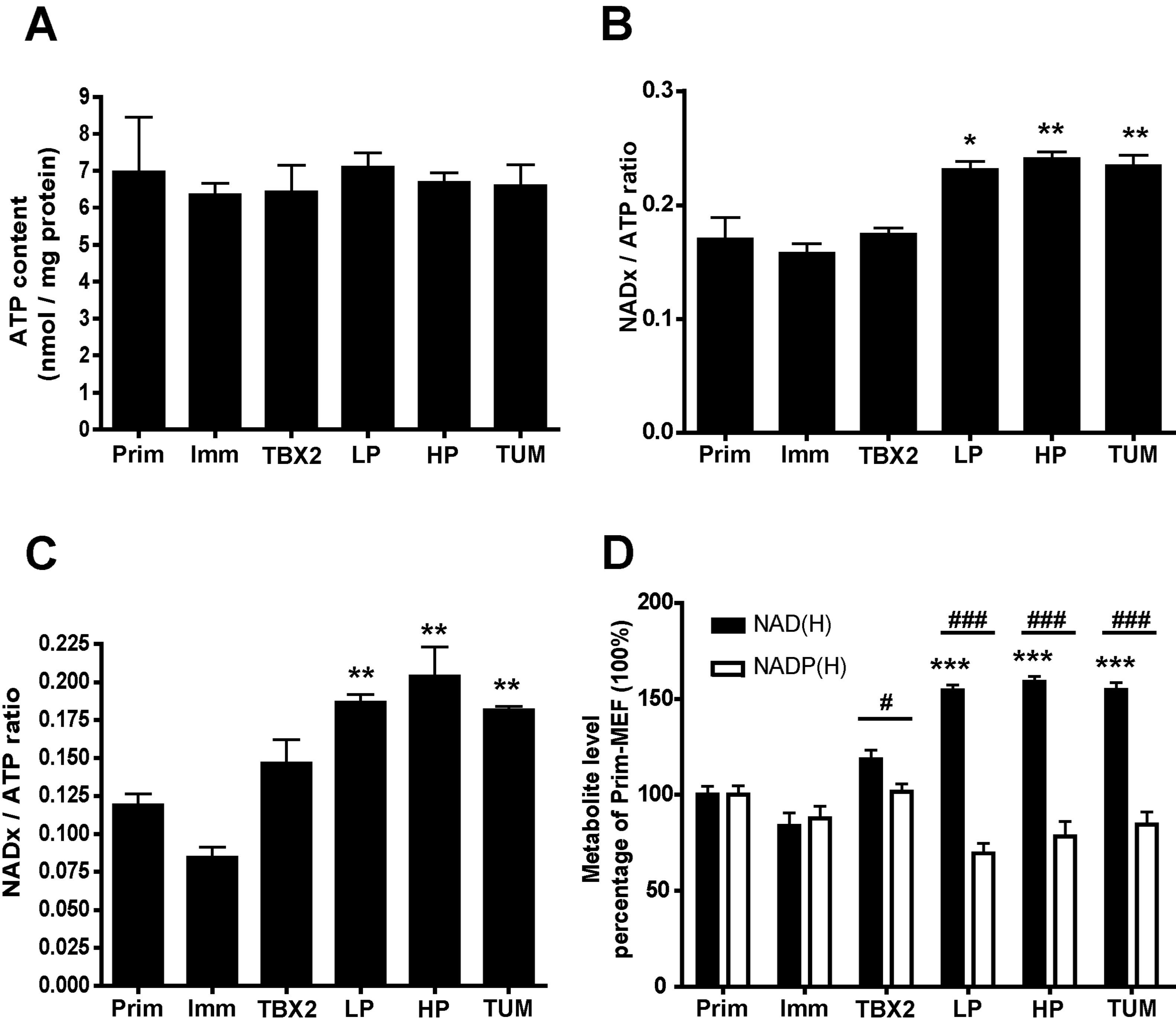
A
ATP content
(nmol / mg protein)
0 1 2 3 4 5 6 7 8 9
Prim Imm TBX2 LP HP TUM
B
NADx / ATP ratio
0.0 0.1 0.2 0.3
*
**
**
Prim Imm TBX2 LP HP TUM
C
NADx / ATP ratio
0.000 0.025 0.050 0.075 0.100 0.125 0.150 0.175 0.200 0.225
**
**
**
Prim Imm TBX2 LP HP TUM
D
Metabolite level
percentage of Prim-MEF (100%)
0 50 100 150 200
NAD(H)
NADP(H)
#
###
###
###
***
***
***
Prim Imm TBX2 LP HP TUM

Figure S7

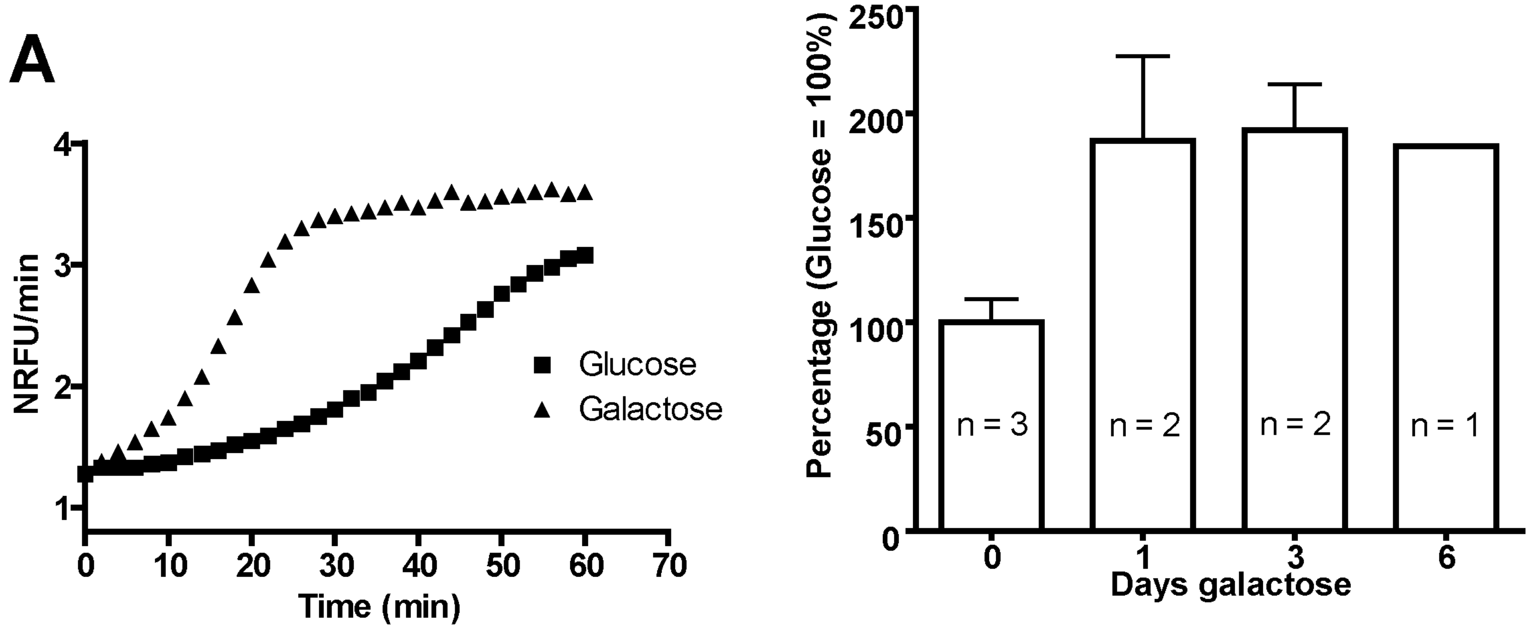
A
NRFU/min
Time (min)
Glucose
Galactose
Percentage (Glucose = 100%)
n = 3
n = 2
n = 2
n = 1
Days galactose

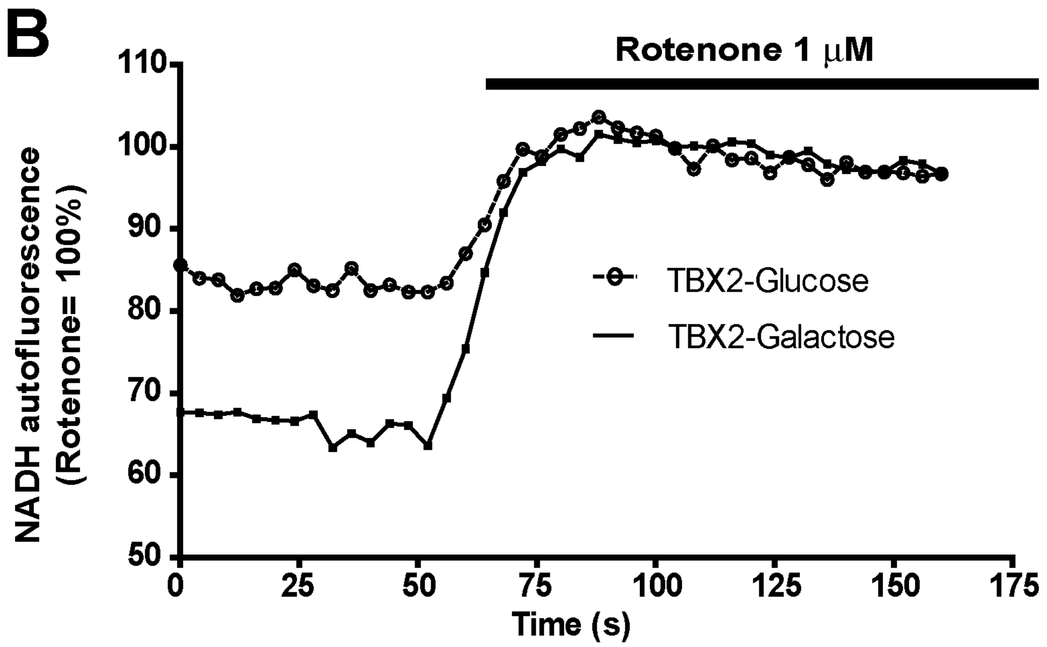
B
Rotenone 1 μM
NADH autofluorescence (Rotenone= 100%)
TBX2-Glucose
TBX2-Galactose
Time (s)

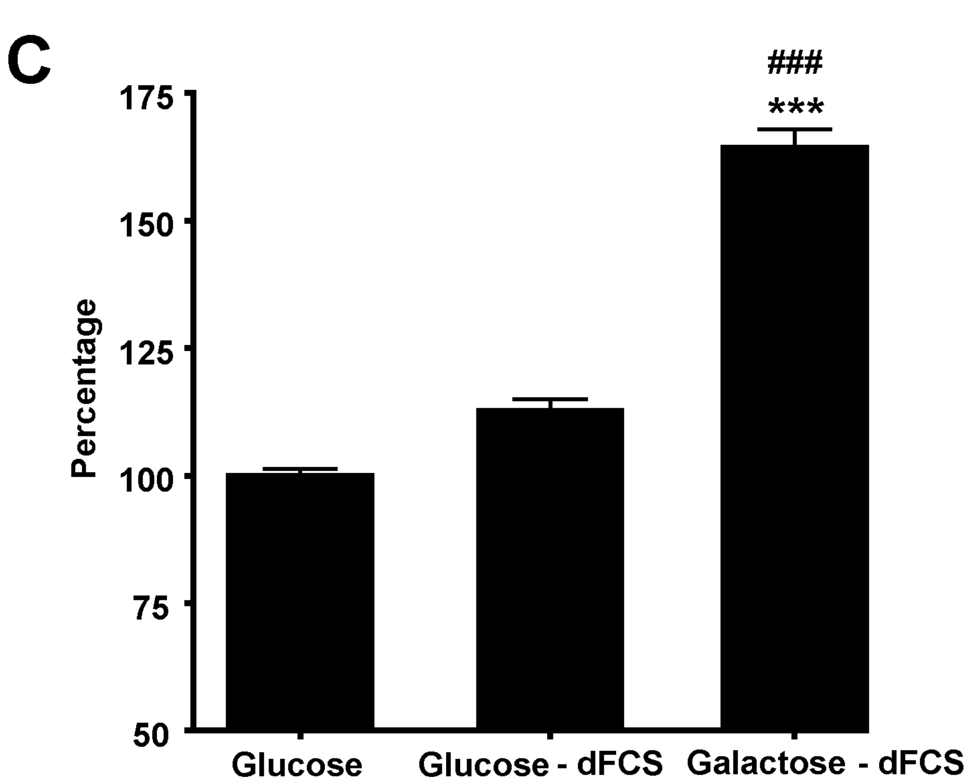
C
###
***
Percentage
Glucose
Glucose - dFCS
Galactose - dFCS
