# Supplementary material for: Increased OXPHOS activity precedes rise in glycolytic rate in H-RasV12/E1A transformed fibroblasts that develop a Warburg phenotype
Source: Mol Cancer. 2009 Jul 31;8:54. doi: 10.1186/1476-4598-8-54 (PMC2734543; doi:10.1186/1476-4598-8-54)
Supplement: Additional file 2 — Additional Material and Methods, Results, Supplemental Figure Legends. File contains supplemental Material and Methods and Results as referred to in the main article. [file 1476-4598-8-54-S2.pdf]

Additional file 2

Increased OXPHOS activity precedes rise in glycolytic rate in H-RasV12/E1A transformed fibroblasts that develop a Warburg phenotype

Ad J.C. de Groof *et al.*

Additional Material and Methods

Retroviral gene transduction - Retroviral vectors pLPC-E1A-ires-H-rasV12 (1) and pBabe-TBX2 (2) were transfected into amphotrophic Phoenix ΦNX-A packaging cells using Lipofectamine 2000 (Invitrogen), and helper-free recombinant retrovirus was produced as described (3, 4). Viral supernatants supplemented with polybrene (20 µg/well; total volume 5 mL) were added to Prim-MEF cells, seeded 1 day prior to infection at 80,000/well in 6-well plates. After 24 hours, viral supernatants were replaced by puromycin selection medium (1 µg/mL), which was applied for a period of 14 days.

Scanning Electron Microscopy - Cells grown in 3.5 cm tissue culture dishes were washed with PBS containing 0.5 mM $MgCl_2$ and 1 mM $CaCl_2$, fixed for 1 hr with 1% glutaraldehyde in 0.1M phosphate buffer pH 7.4 and post-fixed for 30 minutes with 1% osmium tetroxide in 0.1M phosphate buffer pH 7.4. After dehydration in a graded ethanol series, critical point drying (Polaron - Quorum Technologies, Ringmer, UK) and sputter coating with a thin layer of gold, cells were visualized with a JEOL-6310 microscope operated at 15 kV.

Microscopic analysis of cell size - Cells were trypsinized and resuspended in medium supplemented with 10 mM HEPES, pH 7.3. Cell diameter was measured in a Bürker-Türk chamber on a Zeiss Axiovert 35M microscope using a 20x objective with a micrometer (E. Leitz, Wetzlar, Germany) as a reference.

FACS analysis of cell size - Forward scatter and side scatter were determined on cells after paraformaldehyde fixation (2%) on a FACScan (Becton Dickinson) Flow cytometer. Forward scatter principally relates to cell size, and is approximately proportional to cell diameter. However, the forward scatter signal is also partially dependent on cell surface structure and internal granularity, and therefore the relationship between forward scatter and diameter is presumably not perfectly linear. Two independent experiments were performed for each cell line analyzed, with 10,000 cells analyzed in each experiment. The average values of the two experiments are presented.

Soft agar assay - Cells (10,000 per well) in culture medium/0.5% soft agar (Seaplaque, Lonza Biosciences, Breda, The Netherlands) were overlaid on medium/1.0% soft agar plated in 12-well plates. After 8 days, cells were colored using iodonitrotetrazolium chloride (Sigma-Aldrich, Zwijndrecht, The Netherlands). Images were captured 16 hours later and processed to binary images using Metamorph 6.2 software (Molecular Devices Corp., Downingtown, PA). The density of colonies per cm2 was calculated and analyzed in a Student's t-test.

Injection of cells in immune-deficient mice - All procedures involving animals were approved by the Animal Care Committee of the Radboud University Nijmegen Medical Centre, The Netherlands, and conformed to the Dutch Council for Animal Care and the NIH guidelines. One million E1A/H-rasV12 transduced MEFs were injected subcutaneously in 200 µl PBS as xenografts in male BALB/c nu/nu mice. Ras-LP cells

were injected on the left side of each animal, Ras-HP on the right. Tumor dimensions (l*w*h) were measured during 4 weeks or until one of the tumors reached a volume of 1.5 cm^3.

Lysate preparation and Western blot analysis - Cells were rinsed with ice-cold PBS and lysed in buffer containing 50 mM Tris-HCl pH 7.5, 100 mM NaCl, 5 mM $MgCl_2$, 0.5% NP40, 1 mM PMSF, Complete protease inhibitor (Roche), which was further supplemented with phosphatase inhibitors 1 mM Sodium Pyrophosphate, 25 mM NaF, and 0.1 mM Sodium Vanadate (all Sigma-Aldrich) if antibodies to phosphorylated proteins were used. Samples were cleared by centrifugation, frozen in liquid nitrogen and stored at -80ºC. Protein concentration was determined in a Bradford assay. Samples (5-15 µg per lane) in Laemmli sample buffer were separated by SDS-PAGE and transferred to PVDF membranes. Membranes were blocked, and primary antibodies (Additional Table 1) and HRP-conjugated secondary antibodies were applied in buffers and dilutions specified in the supplementary M&M. Peroxidase signals were detected by use of home-made chemiluminescence reagent. Films were scanned for densitometry, and relative protein content in each lane was determined by relating band intensity to the corresponding tubulin signal and Ras-TUM standard samples with 50% and 25% protein (5). Protein quantifications were carried out on two independent lysates and averaged, with Prim-MEF expression levels arbitrarily set to 1.0.

HPLC - In the first set of experiments, sample preparation was performed according to Fiehn[1]. Briefly, cells in three separate 10 cm dishes were each washed with cold PBS and scraped in 2.5 ml MeOH (pH 7.5). Three parallel plates were used for cell counting. The MeOH-scraped cells were spun and the supernatant was collected in a separate tube [A]. The pellet was resuspended in cold 1.0 ml H_2O (pH 7.5), followed by thorough vortexing (20'). Subsequently, 1.0 ml $CHCl_3$ was added and the mixture was vortexed again (20'). After 2' centrifugation at 2,500 rpm at 4ºC, the upper layer was carefully removed, added to tube [A], and 1 ml H_2O was added, followed by another round of intense vortexing and centrifugation. The upper layer was again removed and added to tube [A], which was subsequently frozen in liquid N_2 and stored at -80ºC. The apolar layer was now removed and liquid was evaporated with a continuous stream of N_2 gas. The pellet was resuspended in 0.2 ml H_2O and left on ice for 3-5 hrs. After centrifugation (10', 2,500 rpm), the supernatant was added to the frozen polar extract. The entire extract was allowed to freeze-dry overnight. The next day, 0.2 ml H_2O was added to the freeze-dried product and moderately vortexed. The solution was centrifuged (2', 2,500 rpm), filtered through a Millex-HV 0.45-4 mm filter and analyzed by HPLC immediately.

A downside of the chloroform-methanol extraction method is that quantification of protein levels in the samples is impossible. We therefore calculated internal metabolite ratios and metabolite levels per cell using the cell counts in parallel plates. To allow metabolite quantification per mg cellular protein, a second set of HPLC analyses was carried out with samples that were prepared using the perchlorid acid extraction method (6). Fresh extracts were prepared using cold 0.6 M PCA (perchloric acid) and immediately neutralized by adding 3 M KOH in order to reduce oxidation of metabolites in acid. After spinning down of potassium perchlorate, the supernatant was frozen and freeze-dried in a SpeedVac. The powder was dissolved in distilled water and 50 µl was used for HPLC analysis. The pellet, formed after acid precipitation, was dissolved in 1 M NaOH and used in the Pierce-BCA method (Pierce, Etten-Leur, The Netherlands) to

quantify the protein level of the sample. Experiments were performed on Shimadzu HPLC instrumentation equipped with a Supelcosil LC-18-T, 15 cm x 4.6 mm, 3 μ particles column (Supelco, Sigma-Aldrich). Details of the experimental procedure were described by Stocchi et al. (7). In brief, the mobile phase consisted of Buffer A composed of 0.1 M KH_2PO_4 / 8 mM tetrabutylammonium hydrogen sulphate, pH 6.0 and Buffer B composed of buffer A mixed with methanol (70 : 30), pH 7.2, with a flow rate of 1 ml/min. The gradient program: Start t = 0': 100 % A, t=2.5': 100%A, t=5': 20%B, t=10': 40% B, t=13': 100% B, t = 18': 100 % B, t = 18.1': 100% A, t = 28': stop; injection volume = 50 μl.

[1] Plant Metabolite group, Golm: http://www.mpimp-golm.mpg.de/fiehn/blatt-protokoll-e.html

Microscopic measurement of NAD(P)H autofluorescence - NAD(P)H autofluorescence levels were measured in the experimental setup described by Verkaart et al. (8). Briefly, cells cultured on glass coverslips were placed on an Axiovert 200 M inverted microscope (Carl Zeiss) and transferred to HEPES-Tris-glucose medium. NAD(P)H was excited at 360 nm using a Polychrome IV monochromator (TILL Photonics, Martinsreid, Germany). Emission light was detected through a 415DCLP dichroic mirror and 510WB40 emission filter (Omega Optical, Inc., Brattleboro, VT) onto a CoolSNAP HQ monochrome CCD-camera (Roper Scientific Photometrics, Vianen, The Netherlands). All hardware was controlled using Metafluor 6.0 software (Molecular Devices Corporation, Downingtown, PA, USA). Per coverslip, 10 fields of view were analyzed with comparable image acquisition times (100 ms). All six cell lines were tested in parallel on 3 different experimental days (three coverslips per day) to avoid bias due to day-to-day variation in experimental conditions and excitation light intensity. Statistical significance of differences was tested using the average fluorescence intensity of 3 x 30 regions of interest on an experimental day as experimental unit. The value of the PRIM-MEF cells was arbitrarily set to 100. In a separate set of experiments, the steady state of NAD(P)H was determined as a percentage of maximum NAD(P)H autofluorescence level after inhibition with 1 μM rotenone (Sigma-Aldrich) to obtain information on mitochondrial NAD+/NADH redox couple.

Additional Results

Changes in cell morphology - Cell morphology in Prim-MEF, Imm-MEF and TBX2-MEF populations was largely similar; each pool showing cells that were spread out and had an ultra-flat structure with large lamellipodial processes (Fig.S1A). Introduction of H-RasV12/E1A induced a prominent morphology change and a reduction in size observed from the earliest phase of transformation onwards (Fig.S1B). Diameters of all H-RasV12/E1A cells were between 33-38% of Prim-MEF as measured by microscopic analysis (Table 1), which was corroborated by FACS forward scatter data analysis (Fig.S2A). Remarkably, Ras-HP and Ras-TUM populations formed clusters of cells that remained interconnected during growth, in contrast to the Ras-LP population which remained single-cell (Fig.S1B).

Increased pyridine nucleotide levels in transformed cells - To investigate if adjustments in the balance between glycolysis and OXPHOS were reflected in changes in steady-state levels of total cellular ATP, NAD^+, NADH, NADP, and NADPH levels we used HPLC. ATP levels per mg protein were similar in all cell lines (Fig.S6A). Two independent extraction methods revealed a significant 30-40% increase in total cellular pyridine levels in all RasV12/E1A transformed populations relative to unchanged total cellular ATP level, which gives a fair comparison irrespective of protein content and cell volume (Fig.S6B,C). Fig.S6D shows that the increased pyridine levels can be explained entirely by a significant increase of the NADx (NAD^+ plus NADH) and not NADPx (NADP plus NADPH) levels. The NADx/NADPx ratio in Prim-MEF averages 12.5, which indicates that only a small fraction of NAD is actually converted into NADP. Unfortunately, the data on total NADx metabolite levels and NAD-dependent enzymes did not offer further information on the observed shift in relative contribution of glycolysis and OXPHOS in Ras-LP, Ras-HP and Ras-TUM cells.

Additional References

1. Serrano M, Lin AW, McCurrach ME, Beach D, Lowe SW. Oncogenic ras Provokes Premature Cell Senescence Associated with Accumulation of p53 and p16INK4a. Cell 1997; 88:593-602.
2. Jacobs JJL, Keblusek P, Robanus-Maandag E, et al. Senescence bypass screen identifies TBX2, which represses Cdkn2a (p19ARF) and is amplified in a subset of human breast cancers. Nat Genet 2000; 26:291-9.
3. Kinsella T, Nolan G. Episomal vectors rapidly and stably produce high-titer recombinant retrovirus. Hum Gene Ther 1996; 7:1405-13.
4. Michiels F, Van Der Kammen RA, Janssen L, Nolan G, Collard JG. Expression of Rho GTPases using retroviral vectors
Methods in Enzymology. In: W.E. Balch, Channing J. Der and Alan Hall, editors. Part D: Regulators and Effectors of Small GTPases. Volume 325 ed: Academic Press; 2000. p. 295-302.
5. de Groof A, Oerlemans F, Jost C, Wieringa B. Changes in glycolytic network and mitochondrial design in creatine kinase-deficient muscles. Muscle Nerve 2001; 24:1188-96.
6. Bernocchi P, Ceconi C, Cargnoni A, Pedersini P, Curello S, Ferrari R. Extraction and assay of creatine phosphate, purine, and pyridine nucleotides in cardiac tissue by reversed-phase high-performance liquid chromatography. Anal Biochem 1994; 222:374-9.
7. Stocchi V, Cucchiarini L, Canestrari F, Piacentini MP, Fornaini G. A very fast ion-pair reversed-phase HPLC method for the separation of the most significant nucleotides and their degradation products in human red blood cells. Anal Biochem 1987; 167:181-90.
8. Verkaart S, Koopman WJH, Cheek J, et al. Mitochondrial and cytosolic thiol redox state are not detectably altered in isolated human NADH:ubiquinone oxidoreductase deficiency. Biochimica et biophysica acta 2007; 1772:1041-51.

Additional Figure Legends

Figure S1 - Morphological appearance of cell lines with different oncogenic potential (Scanning Electron Microscopy)

A: Primary and immortalized cells. Magnification 500x; scale bar 10 µm.
B: H-RasV12/E1A transformed cells. Magnification 1000x; scale bar 10 µm
H-RasV12/E1A-transformed cells change from flat to spherical cells. The transition from the Ras-LP to the Ras-HP stage is characterized by increased formation of cell clusters.

Figure S2 - Cell diameter and protein content

A: Comparison of cell diameter measurements performed by microcopy analysis or by FACS Forward scatter. Forward scatter data (mean ± SD) are average of 2 independent experiments. Microscopy data (mean ± SD) correspond to the data in Table 1. The two methods give exactly similar results.
B: Protein concentration (mean ± SEM; n=3, triplicates per individual experiment) in ng per cell measured in PCA extracts for HPLC (see Fig.S6). *:p<0.05; **:p<0.01; ***:p<0.001 compared to Prim-MEF (one-way ANOVA/Bonferroni). Protein concentration in ng per cell decreases, as does cell volume (calculated from data in (A)). This implicates that a decrease in cell volume does not result in an increased protein density per cell volume.
C: Protein concentration (mean ± SEM) in ng per pL cell volume. Although the protein concentration per pL appears decreased in immortalized cells, and increased in H-RasV12/E1A transformed cells, none of the comparisons between primary cells and the other populations reached statistical significance.

Figure S3 - Lactic acid production and oxygen consumption per cell

A: Lactic acid production in pmoles/cell (mean ± SEM; n=6 in all cell populations, except Ras-HP (n=8) and Ras-TUM (n=7)). These values were measured in media samples taken from the cell cultures used to obtain the cell doubling time data in Table 1.
*:p<0.05; **:p<0.01; ***:p<0.001 compared to Prim-MEF (one-way ANOVA/Bonferroni)
##: p<0.01; ###:p<0.001 for Ras-LP – Ras-HP – Ras-TUM intercomparisons (one-way ANOVA/Bonferroni).
B: Oxygen consumption in NRFU/min/cell measured in BD oxygen biosensor plates (mean ± SEM). Statistics: see A.

Figure S4 - Sensitivity of oxygen consumption to PCCP and inhibitors

A: Oxygen consumption measured in oxygen biosensor plates. Filled bars represent oxygen consumption in normalized relative fluorescence units (NRFU) per min per cell (see Fig.S4B). Open bars represent oxygen consumption in the presence of FCCP (2 µM) in parallel experiments.
B: Oxygen consumption under normal conditions in oxygen biosensor plates is presented here as a percentage of the FCCP-induced maximum respiratory activity (data from A). *:p<0.05 compared to Prim-MEF (one-way ANOVA/Bonferroni).
C: Application of the Complex I inhibitor oligomycin (2.5 µM) almost completely blocks oxygen consumption in oxygen biosensor assays in all cell populations (open bars).

Oxygen consumption is presented as a percentage of normal activity (filled bars) that was measured in parallel experiments.

D: Application of the Complex III inhibitor myxothiazol (1 μM) completely abolishes oxygen consumption in oxygen biosensor assays in H-RasV12/E1A populations. Application of extracellular NADH (1 μM), which blocks membrane oxygen consumption, only marginally affects oxygen consumption in these cell populations. Other cell populations were not analyzed. Oxygen consumption is presented as a percentage of normal activity that was measured in parallel
experiments.

Figure S5 - Western Blots for enzymes in glycolysis (panel A), TCA cycle/OXPHOS (panel B), and NAD^+ metabolism (panel C)

Numbers between brackets behind the protein name indicate analyses that have been performed on the same membrane (f.e. tubulin controls). The appropriate bands were cut from the original image and optimized using the "auto-levels" option in Photoshop CS2. Original images are available upon request.

Arrows indicate significant upregulation of protein levels for TKT, MDH1 and Nampt.

Figure S6 - Cellular metabolite content measured by HPLC

A: ATP levels per mg protein determined in PCA cell extracts used for HPLC analysis (mean + SEM; n=3 per cell population). These levels are equal in all cell populations.

B, C: Increase of NADx/ATP ratio in Ras-LP, Ras-HP and Ras-TUM cells. Total NADx (NAD^+, NADH, NADP, NADPH) and ATP levels were determined by HPLC in Fiehn (**B**, n=4, triplicates per individual experiment) and PCA (**C**, n=3, triplicates per individual experiment) cell extracts. These values are independent of cell volume and cell protein content.

*: $p<0.05$; **: $p<0.01$, compared to Prim-MEF (one-way ANOVA/Bonferroni)

D: Increase in NADx content is caused by increased NAD^+ plus NADH levels [NAD(H)], not NADP plus NADPH [NADP(H)] levels. Graph presents NAD^+ plus NADH levels relative to ATP levels (filled bars) and NADP plus NADPH levels relative to ATP levels (open bars). The average metabolite / ATP ratio of Prim-MEF was set to 100% to allow comparison of metabolites with different cellular concentrations. Graph shows combined results of Fiehn and PCA analysis (n=7, triplicates per individual experiment).

***: $p<0.001$ compared to Prim-MEF (one-way ANOVA/Bonferroni)

#: $p<0.05$; ###: $p<0.001$ in comparison NAD vs NADP levels in Student's t-test.

Prim-Mef NAD(H) concentration: 0.71 nmol/mg protein; NADP(H) concentration 0.07 nmol/mg protein.

Figure S7 - Oxygen consumption, NADH autofluorescence and ROS production

A: Oxygen consumption in TBX2-MEF in glucose and galactose medium. Left panel shows traces of representative individual experiment (6 days galactose) with equal amounts of cells per well. Right panel shows cumulative data, with the oxygen consumption in glucose medium arbitrarily set to 100%. Cells grown in galactose medium consume about two-fold more oxygen.

B: Representative recordings of NADH autofluorescence in mitochondria of TBX2-MEF cells before and after rotenone application. Cells were grown in 25 mM glucose or 10 mM galactose medium/dialyzed FBS. TBX2-MEF cultured on 10 mM galactose medium/dialyzed FBS, which show increased oxygen consumption levels, show a relatively lower level of NADH autofluorescence.

C: Superoxide levels in TBX2-MEF expressed as fluorescence signal of HEt oxidation products. Superoxide levels were determined using FACS analysis. Superoxide levels are significantly increased in TBX2 cells cultured on galactose. ***/###: $p<0.001$ in one way Anova compared to glucose control.

Additional Table S1 - Antibodies used for quantitative Western Blot analysis

abbreviation	directed against	supplier/source	dilution	blocking/incubation
ALD	aldolase, rabbit muscle	Rockland, Gilbertsville, PA: 100-1141	1 : 5000	3% fat-free milk / TBST
GAPDH	glyceraldehyde 3 phosphate dehydrogenase, human	Cell Signaling Technology, Danvers, MA: 14C10 (#2118)	1 : 1000	3% fat-free milk / TBST
PK-M2	pyruvate kinase M2, human	Cell Signaling Technology, Danvers, MA: #2118	1 : 1000	3% fat-free milk / TBST
LDH	lactate dehydrogenase, rabbit muscle	Rockland, Gilbertsville, PA: 100-1173	1 : 2500	3% fat-free milk / TBST
VDAC / Porin 31 HL	voltage-dependent anion channel, (anti porin 31HL, Human)	Calbiochem, La Jolla, Ca: 529532	1 : 5000	3% fat-free milk / TBST
B39	Complex I subunit 39 kDa	Molecular Probes, Leiden, the Netherlands	1 : 1000	3% fat-free milk / TBST
PDH-E2	pyruvated dehydrogenase complex, human	Mitoscience, Eugene, OR: MSP02	1 : 1000	3% fat-free milk / TBST
PDH-E1a	pyruvated dehydrogenase complex, human	Mitoscience, Eugene, OR: MSP02	1 : 1000	3% fat-free milk / TBST
MDH1	malate dehydrogenase, soluble, human	Abnova, Niehu, Taiwan	1 : 5000	3% fat-free milk / TBST
MDH2	mitochondrial malate dehydrogenase, pig heart	Rockland, Gilbertsville, PA: 100-601-145	1 : 5000	3% fat-free milk / TBST
NAMPT	NAMPT /pre-B-cell colony enhancing factor 1, human	Bethyl laboratories, Montgomery, TX: A300-372A	1 : 1000	3% fat-free milk / TBST
Tubulin	beta-tubulin, mouse	Developmental studies hybridoma bank, Iowa City, IA: E7	1 : 5000	3% fat-free milk / TBST
